# Updated checklist of *Poa* in the Iberian Peninsula and Balearic Islands

**DOI:** 10.3897/phytokeys.103.26029

**Published:** 2018-07-10

**Authors:** Ana Ortega-Olivencia, Juan A. Devesa

**Affiliations:** 1 Área de Botánica, Facultad de Ciencias, Universidad de Extremadura, Avenida de Elvas, s.n., 06006 Badajoz, Spain; 2 Departamento de Botánica, Ecología y Fisiología Vegetal, Facultad de Ciencias, Universidad de Córdoba, Campus de Rabanales, Edificio José Celestino Mutis, Ctra. de Madrid km. 396 A, 14014 Córdoba, Spain

**Keywords:** Checklist, *Flora iberica*, Gramineae, Poaceae, *Poa*, Portugal, Spain, taxonomy, typification

## Abstract

Based on our study of 4,845 herbarium sheets of the genus *Poa* from the area covered by *Flora iberica*, namely, the Iberian Peninsula and the Balearic Islands, we recognise 24 taxa (17 species, 1 subspecies and 8 varieties), mostly perennials. Most of these taxa have wide global and/or European distributions, while two (*P. legionensis* and P. minor subsp. nevadensis) are Spanish endemics and two have restricted distributions (*P. ligulata*, Iberia–North Africa; *P. flaccidula*, Iberia–North Africa and the Balearic Islands, extending to Provence, France). We have studied the original publications of more than 225 names considered as synonyms, with those more historically cited in *Flora iberica* taken into account in this paper; a total of 26 are new synonyms. The following names are typified: P. alpina var. involucrata Lange, P. annua var. lanuginosa Sennen, P. minor subsp. nevadensis Nannf., *P. paui* Font Quer, *P. sulcata* Lag. and P. trivialis var. flaccida Willk. ex J.J. Rodr. We include *P. compressa* L. in the flora of Portugal for the first time and present detailed illustrations of three very interesting taxa (*P. legionensis*, P. minor subsp. nevadensis and *P. ligulata*). In addition to a general species key, we provide the following information for each taxon: synonyms, types, typification, the most relevant iconography, regional flowering time, regional and general distribution and, as supplementary material, the number of sheets examined and a list of selected materials.

## Introduction

The genus *Poa* L., included within subfamily Pooideae, supertribe Poodae and subtribe Poinae (Soreng et al. 2015), is considered to be monophyletic. This monophyly is supported by analyses of plastid and nuclear DNA markers (Gillespie et al. 2008). In addition, evidence for reticulation between this genus and other genera in Poinae has been uncovered; the same is true within the genus itself (e.g. *P. annua*) (Soreng et al. 2010). The genus comprises approximately 550 annual and perennial species (Soreng et al. 2017) of cosmopolitan distribution, primarily in cold and temperate regions. Most species are polyploids, but 9% are diploids, with an additional 4%–6%, mostly in Europe and rarely in Asia, having both diploid and polyploid populations (Soreng et al. 2010, Giussani et al. 2016). Many species are important weeds (e.g. *P. annua*), while others are cultivated for forage (e.g. *P. pratensis*) or used in pastures (e.g. *P. trivialis, P. alpina* and *P. bulbosa*) or lawns and golf courses (e.g. *P. nemoralis* and *P. pratensis*) (Watson and Dallwitz 1992).

The genus is characterised by a great diversity of sexual systems and its species can be strictly hermaphroditic, the most common reproductive system, or diclinous (Giussani et al. 2016). Apomictic reproduction by seeds, either facultative or obligate, is common in some species (reviewed in Soreng and Peterson 2012). The production of pseudoviviparous/bulbiferous spikelets occurs in some species, such as *P. bulbosa* and, to a lesser extent, *P. alpina*.

The first taxonomic treatment of *Poa* on the Iberian Peninsula, by Willkomm (1870), was remarkable: he recognised 15 species and numerous varieties in the territory of Spain and later increased this total by two (Willkomm 1893). In the 20^th^ century, 19 of the 53 species and subspecies recognised by Edmonson (1980) in Europe were included in *Flora iberica*, a publication also encompassing the Balearic Islands. Of these, slightly less than half were listed for Portugal (see also Franco and Rocha 1998). The latest revision to *Poa* on the peninsula was carried out by Hernández Cardona (1978), who basically followed Edmonson. In this revision, Hernández Cardona also defined a new section (*Flaccidula* Á.M. Hern.) to accommodate *P. flaccidula* and recognised 19 species and subspecies plus two varieties.

In a recently completed revision of the genus *Poa* for a future volume (XIX) of *Flora iberica*, we recognised 18 species and subspecies and 8 varieties. The main aim of the present paper was to present an updated checklist of the genus. The information provided includes a general key to accepted taxa as well as a list of their most important synonyms, many of which are unknown outside of the Iberian Peninsula because they are found only on herbarium sheets or published in works of limited distribution. Some of the synonyms and an accepted name are typified and updated information on the ecology and flowering characteristics of each taxon in the covered territory is given along with its regional and worldwide distribution.

## Methods

The taxonomic classification scheme followed in this paper, which begins with the type species *P. pratensis*, reflects currently understood relationships amongst recognised sections in the genus. An infrageneric classification of accepted species of *Poa* in the Iberian flora is also presented in the Results section.

**Figure 1.**
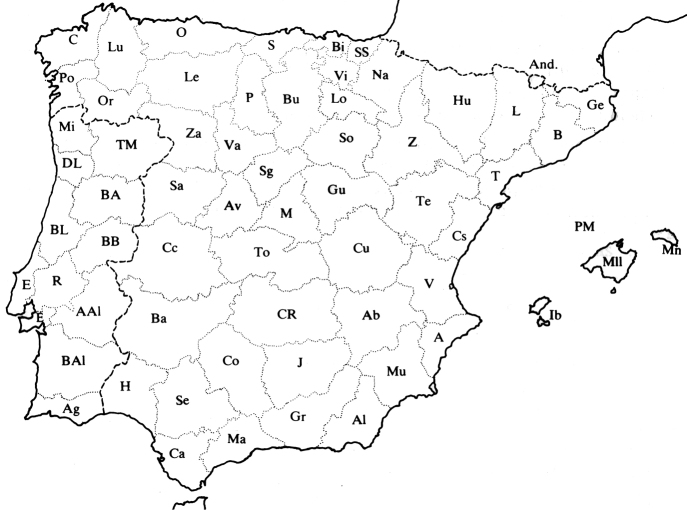
Map of distribution of the provinces covered by *Flora iberica* (http://bibdigital.rjb.csic.es/spa/Libro.php?Libro=476&Pagina=27).

We reviewed 4,845 sheets housed in the following herbaria: BC, BC-Sennen, C-Lange, COI, COI-Willk., G-Boiss., GDA-GDAC, HGM, HSS, JACA, MA, MAF, MGC, SALA-SALAF, SEV, UPP-Nannf. and UNEX (acronyms according to Thiers, continuously updated). We studied the most important synonyms of each accepted name and consulted the original publications, with a special focus on names directly related to the territory covered by *Flora iberica*. For each accepted taxon, we recorded synonyms, types (type protologue) and, in some cases, the typification. After studying the herbarium sheets, we obtained updated information on flowering phenology and the ecology of the area. We also researched the worldwide distribution of each taxon and its presence or absence in each province covered by *Flora iberica*, including the territories of Andorra (And.), Portugal (Port.) and continental Spain (Spa.) plus the Balearic Islands. In the taxonomic treatment that follows, those provinces are ordered alphabetically using the same abbreviations given in *Flora iberica* (http://www.floraiberica.es/; see Fig. 1). If the name of a province appears in parentheses, a bibliographic citation exists but no herbarium sheet was studied to confirm it, while a question mark indicates that the bibliographic citation is not entirely reliable. A selected list of herbarium sheets studied from each province is provided in the Suppl. material 1. Finally, some observations are included as explanatory notes for most species and subspecies.

## Results and discussion

Currently, a total of 24 taxa are recognised: 17 species, 1 subspecies and 8 varieties. All are perennials except for *P. annua* and *P. infirma*, which are annuals.

### Key to the species of *Poa* in Flora iberica

**Table:** 

1	Plant annual, sometimes multicaulous	**2**
–	Plant perennial, cespitose, rhizomatous and/or stoloniferous	**3**
2	Spikelets (1.6−)4−7.7(−9) mm; anthers (0.6−)0.7−1.3 mm, much longer than wide; caryopsis 1.3−2.1 mm	**12. *P. annua***
–	Spikelets 2.8–4.8 mm; anthers 0.2−0.4(−0.6) mm, the same as or slightly longer than the width; caryopsis 1−1.4 mm	**13. *P. infirma***
3	Plant cespitose, without rhizomes and almost always without stolons, usually with basal bulbils and/or stems with thickened bases covered by old sheaths, these often split in fibres	**4**
–	Plant cespitose, with rhizomes and/or stolons, lacking bulbils or strongly thickened stem bases surrounded by old sheaths or, if present, then with the ligule of basal leaves 0.15−0.4(−0.8) mm, truncate	**9**
4	Lemma with 5 prominent veins and base very hairy, with hairs longer than the width of the lemma	**1. *P. pratensis***
–	Lemma with 5 inconspicuous veins and base glabrous or with hairs much shorter than the width of the lemma	**5**
5	Plant without stolons, bulbils or stems thickened at the base, glaucous; glumes lanceolate	**5. *P. glauca***
–	Plant with short stolons rooting at the nodes or with bulbils or thickened stem bases covered by old sheaths, green or glaucous; glumes lanceolate or the lowermost one narrowly subulate	**6**
6	Plant cespitose, rarely with short stolons rooting at the nodes, without basal bulbils or thickened stem bases; lower glume narrowly subulate	**6. *P. nemoralis***
–	Plant cespitose, with basal bulbils and/or strongly thickened stem bases covered by old fibrous sheaths; lower glume lanceolate or ovate-lanceolate	**7**
7	Basal and shoot leaves with ligule 0.3−2(−3.8) mm, the oldest 0.3–0.7 mm, ± truncate, blade (0.7−)1.5−4.5(−7.5) mm wide, flat	**17. *P. alpina***
–	Basal and shoot leaves with ligule 2−10.5 mm, ± oblong or triangular, often laciniate in acute triangular segments, blade 0.4−3.5(−4) mm wide, flat, conduplicate or convolute	**8**
8	Plant with basal bulbils, frequently also present in the inflorescence; basal and shoot leaves filiform or linear, less frequently lanceolate; ligule membranous-hyaline; tuft concoloured, green or brown	**15. *P. bulbosa***
–	Plant without basal bulbils or bulbils in the inflorescence; basal and shoot leaves linear or lanceolate; ligule pearly white; tuft bicoloured, green and white because of the brightness of the ligules	**16. *P. ligulata***
9	Branches of the inflorescence and/or spikelet peduncles smooth or nearly so, glabrous	**10**
–	Branches of the inflorescence and/or spikelet peduncles antrorse-scabrid	**14**
10	Ligule of the basal leaves 0.15−0.7(−0.8) mm, truncate	**11**
–	Ligule of the basal leaves 0.6−3.4(−4.3) mm, more or less ovate or triangular-ovate, not truncate	**12**
11	Upper leaves with ligule 0.3−0.6(−0.8) mm, truncate-dentate; lemma base with hairs longer than the width of the lemma; plant cespitose-rhizomatous	**2. *P. legionensis***
–	Upper leaves with ligule 0.7−2.5(−4.7) mm, more or less truncate or ovate, sometimes split into 2 or several parts; lemma base glabrous; plant densely cespitose	**17. *P. alpina***
12	Palea with keels appressed-hairy or ciliate, rarely smooth and glabrous; anthers (1.2−)1.6−2.1 mm; branches of the inflorescence patent or reflexed after anthesis; upper flower of the spikelet female	**14. *P. supina***
–	Palea with keels antrorse-scabrid, never appressed-hairy or ciliate; anthers 0.7−1.7 mm; branches of the inflorescence erect or erect-patent; upper flower of the spikelet hermaphroditic	**13**
13	Anthers 0.7−1.2 mm; branches of the inflorescence 0.15−0.2 mm in diameter, sulcate, ± rigid, glabrous and smooth; spikelets with peduncle glabrous, smooth, having 2−4 flowers	**8. *P. laxa***
–	Anthers 0.8−1.7 mm; branches of the inflorescence c. 0.05−0.15 mm in diameter, not sulcate, ± flexuous, glabrous and smooth, sometimes very loosely antrorse-scabrid; spikelets with peduncle glabrous or sometimes laxly antrorse-scabrid, having 4−7 flowers	**9. *P. minor***
14	Stems compressed	**15**
–	Stems not or only slightly compressed	**21**
15	Lemma and palea with hairy surfaces between veins	**11. *P. flaccidula***
–	Lemma and palea with glabrous surfaces between veins	**16**
16	Ligule of the upper leaf lanceolate, generally acute, longer than the width of the blade	**10. *P. trivialis***
–	Ligule of the upper leaf truncate, obtuse, shorter or subequal to the width of the blade	**17**
17	Lemma with conspicuous veins	**18**
–	Lemma with inconspicuous veins	**19**
18	Stems with base 2.5−9 mm wide, usually very compressed; lemma with 5 glabrous veins; blade of the basal leaves (3.5−)5.5−11.2 mm wide, lanceolate or oblong-lanceolate	**4. *P. chaixii***
–	Stems with base 1.5−2.5(−3.5) mm wide, slightly compressed; lemma with 5 veins, the central and marginal ones appressed-hairy; blade of the basal leaves 0.5−3.8 mm wide, lanceolate, linear or setaceous	**1. *P. pratensis***
19	Usually with two keels in the stems; glumes lanceolate or ovate-lanceolate, ± convergent; spikelet with rachilla usually glabrous; inflorescence with 1−2(−5) branches in the basal node, short and appressed	**7. *P. compressa***
–	Without keels in the stems; glumes subulate or lanceolate-subulate, straight and ± divergent; spikelet with rachilla glabrous or pubescent; inflorescence with 2−5 branches in the basal node, short or long, erect, rarely erect-patent or non-appressed on the axis	**20**
20	Plant rhizomatous; leaves of the shoots usually distichous; spikelets 4.5−7(−7.6) mm; lower glume (2.8−)3.2−4.2 mm, lanceolate	**3. *P. cenisia***
–	Plant without rhizomes, sometimes with short stolons; leaves of the shoots non-distichous, sparse; spikelets 3−4.8(−5.5) mm; lower glume 2−3.5 mm, subulate	**6. *P. nemoralis***
21	Lemma and palea with surfaces between veins hairy	**11. *P. flaccidula***
–	Lemma and palea with surfaces between veins glabrous	**22**
22	Ligule of the upper leaf usually lanceolate, acute or acuminate, longer than the width of the blade; stems usually retrorse-scabrid around the nodes; plant stoloniferous; callus of lemma with hairs longer than the width of the lemma, very rarely glabrous	**10. *P. trivialis***
–	Ligule of the upper leaf ovate or oblong, obtuse, truncate or dentate, much shorter or subequal to the width of the blade; stems smooth around the nodes; plant rhizomatous, rarely with stolons; callus of lemma without hairs or with hairs larger or smaller than the width of the lemma	**23**
23	Plant cespitose, sometimes with short stolons; glumes subulate or lanceolate-subulate, glossy; lemma with base glabrous or with hairs of much shorter length than the width of the lemma; ligule of the upper leaves 0.2−0.6(−0.8) mm	**6. *P. nemoralis***
–	Plant rhizomatous; glumes lanceolate, dull; lemma with base hairy, with hairs shorter or longer than the width of the lemma; ligule of the upper leaves usually 1−3.1 mm	**24**
24	Plant ± glaucous; shoots usually with leaves distichous; culms usually scabrous below nodes; glumes subequal, ± straight or slightly converging with each other, 3-veined; lemma with 5 inconspicuous veins, with hairs at the base shorter or longer than the width of the lemma; palea with keels antrorsely-scaberulous or ciliolate, often something curly in the basal area	**3. *P. cenisia***
–	Plant usually green; shoots usually with non-distichous leaves; culms smooth below nodes; glumes unequal, curved or converging with each other, the lower with 1 or 3 veins; lemma with 5 conspicuous veins, with hairs at the base longer than the width of the lemma; palea with keels antrorsely-scaberulous	**1. *P. pratensis***

### Checklist of *Poa* in the Iberian Flora

The species of *Poa* present in *Flora iberica* are classified into three subgenera and nine sections as indicated below. These taxonomic placements are provisional because some taxa (indicated by *) have not yet been subjected to DNA sequencing:

Poa subgen. Poa supersect. Poa sect. Poa: P. pratensis, *P. legionensis, P. cenisia; supersect. Homalopoa (Dumort.) Soreng & L.J. Gillespie sect. Homalopoa: *P. chaixii*.

Poa subgen. Stenopoa (Dumort.) Soreng & L.J. Gillespie sect. Stenopoa: *P. glauca, P. nemoralis*; sect. Tichopoa Asch. & Graebn.: *P. compressa*; sect. Oreinos Asch. & Graebn.: *P. laxa, P. minor*; sect. Pandemos Asch. & Graebn.: *P. trivialis*; *sect. Flaccidula Á.M. Hern.: *P. flaccidula.

Poa subgen. Ochlopoa (Asch. & Graebn.) Hyl. sect. Micrantherae Stapf: *P. annua, P. infirma, P. supina*; sect. Arenariae Stapf: *P. bulbosa*; sect. Alpinae (Nyman) Stapf: *P. ligulata, P. alpina*.

The sequence of species in this checklist is not alphabetical, but instead starts with sect. Poa because that section includes the type species; species are then ordered according to phylogenetic relationships, an arrangement more or less the inverse of that adopted by other authors, i.e. from more derived clades to those in a more basal position (see Gillespie et al. 2008; Soreng et al. 2010, 2017).

#### 1. Poa pratensis L., Sp. Pl. 67. 1753 subsp. pratensis

Taxon classificationPlantaePoalesPoaceae

Poa angustifolia var. pratensis (L.) Simonkai, Enum. Fl. Transsilv. 580. 1886.
Paneion pratense (L.) Lunell, Amer. Midl. Nat. 4: 222. 1915.
**Ill.** var. pratensis [Soreng and Peterson (2012: 67, fig. 18C–J, sub P. pratensis subsp. pratensis); Devesa (1987: 261, sub P. pratensis)]; var. minor [Soreng and Peterson (2012: 67, fig. 18 A-B, sub P. pratensis subsp. irrigata )]; var. angustifolia [Ruiz (1991: 29, lam. II, sub P. angustifolia]; Soreng and Peterson (2012: 66, fig. 17H–I, sub P. pratensis subsp. angustifolia)].

##### Type

“Habitat in Europae pratis fertilissimis”. Typus: Russia, Prov. Sanct-Petersburg, 5 km australi-occidentum, versus a st. viae ferr. Mga. pratulum ad ripam dextram fl. Mga, 26 Jun 1997, N. N. Tzvelev N-257 (type conserved, designated by Soreng and Barrie 1999, pg. 157: BM-000576302; isolectotypes: B, C, CAN, CONC, H, K, KW, L, LE, LIV, MA, MO, MW, NSW, P, PE, PR, S, SI, TNS, US, W).

##### Flowering.

April-August (September).

##### Ecology.

Grasslands at edges of watercourses, ravines, ponds and alpine wetlands (“borreguiles”), walls, wet soils on slopes, ditches, cultivated fields, clearings surrounded by pines, holm oaks, Portuguese oaks and other oaks; edaphically indifferent; 0−2400 m a.s.l.

##### Distribution.

Eurasia, N Africa and Macaronesia (Azores, Madeira and Canary Islands); introduced in N, C and S America and Australia. Scattered throughout much of the Iberian Peninsula and Balearic Islands. **And. Spa.**: A Ab Al Av B Bu C Cc Co CR Cs Cu Ge Gr Gu Hu J L Le Lo Lu M Ma Mu Na O Or P PM[Mll] (Po) S Sa Sg So SS (T) Te To V Va Vi Z Za. **Port**: AAl (BA) (BB) (BL) DL (E) Mi TM.

##### Notes.

*Poa pratensis* is one of the most polymorphic taxa in the genus for a variety of reasons: its great morphological and cytological variation, the predominance of agamospermy, its vegetative propagation and wide distribution, the latter due in part to its introduction into many parts of the world for use on lawns, as fodder or for soil stabilisation (Soreng and Barrie 1999). At least 220 crop varieties are recognised (Stoneberg Holt et al. 2004).

In the territory covered by *Flora iberica*, three patterns of variation are recognised. Plants with scarcely any extravaginal shoots and possessing basal-leaf ligules with scattered or sometimes entangled apical hairs up to 0.4 mm and 0.2–0.5 mm on the back correspond to ***Poa pratensis*** var. ***minor*** Wahlenb., Fl. Upsal. 33. 1820. [Type: “Hab. in pratis et pascuis fertilibus plerisque frequenter”; *Poa humilis* Ehrh., Beitr. Naturk. 6: 84. 1791, nom. nud.; *P. humilis* Ehrh. ex Hoffm., Deutschl. Fl. 1: 45. 1800, type: “In cultis, ad vias; fl. Apr. Sept.”; *P. subcaerulea* Sm., Engl. Bot. 14, lam. 1004. 1802, basion., type: “Gathered in Anglesea by the Rev. H. Davies, flowering in June”; *P. depressa* J. Presl & C. Presl, Fl. Čech. 20. 1819, type: “Summa Sudetorum cacuminal”; P. pratensis var. latifolia Weihe ex Mert. & W.D.J. Koch, Deutschl. Fl. 1(2): 612. 1823, type: “Auf dürren sandigen Hügeln, auf magern Grasplätzen und auf den Triften hoher Gebirge bleibt”; P. pratensis var. subcaerulea (Sm.) Sm., Engl. Fl. 1: 126 (1824); P. pratensis var. humilis (Ehrh. ex Hoffm.) Ehrh. ex Spenn., Fl. Friburg. 1: 130. 1825; P. pratensis subsp. latifolia (Weihe ex Mert. & W.D.J. Koch) Schübl. & G. Martens, Fl. Würtemberg 77. 1834; P. pratensis var. depressa (J. Presl & C. Presl) Opiz, Seznam Rost. Kvet. Cesk. 76. 1852; P. pratensis var. maritima Corb., Nouv. Fl. Normandie 655. 1894, type: “Sables maritimes et pelouses du littoral. C.”; *P. irrigata* Lindm., Bot. Not. 1905: 73, 88. 1905, type: “Hab. in uliginosis, pratis et viarum marginibus irrigatis, fossis graminosis, solo abiegnorum muscoso humido, haud raro in pratis litoralibus, hinc inde in pascuis solo duriore turfoso. Vidi specimina typica ex Ölandia (Borgholm), .....”; P. irrigata f. rigens Lindm., Bot. Not. 1905: 90. 1905, type: “Hab. In Lapponia”; *P. pratensis* “race” *subcaerulea* (Sm.) Rouy, Fl. France 14: 283. 1913; P. pratensis subsp. irrigata (Lindm.) H. Lindb., Exsicc. (Pl. Finland.) 2: 20. 1916; P. pratensis subsp. subcaerulea (Sm.) Hiitonen, Suomen Kasvio 205, fig. 5. 1933]. This variety is known from N and C Europe (introduced in N America) and also appears on N and SW portions of the Iberian Peninsula [**Spa.**: B H Na S SS (Z)], where it is found on grasslands, nitrified dunes and mountainous limestone rocks [0−1380 m a.s.l. May to September].

Two varieties with abundant extravaginal shoots and basal-leaf ligules without hairs or with hairs that are smaller than 0.15 mm, are recognised, although plants having intermediate characteristics are also frequently present. ***Poa pratensis*** var. ***pratensis*** [*Poa glabra* Ehrh., Beitr. Naturk. 6: 82. 1791, nom. nud.; P. pratensis var. anceps Gaudin, Agrost. Helv. 1: 215. 1811, type: “Hab. in paludibus torfaceis. Schleicher. Perennis”; P. pratensis subsp. anceps (Gaudin) Lej. & Courtois, Comp. Fl. Belg. 82. 1828; *P. pratensis* α *vulgaris* Gaudin, Fl. Helv. 1: 258. 1828, nom. superfl.; *P. anceps* (Gaudin) Hegetschw., Fl. Schweiz 81. 1838, nom. illeg., non *Poa anceps* G. Forst., Fl. Ins. Austr. 8. 1786; P. angustifolia subsp. anceps (Gaudin) K. Richt., Pl. Eur. 1: 87. 1890; *P. pratensis* “race” *compressiformis* Rouy, Fl. France 14: 283. 1913, type: “HAB. — Prairies ombragées, tourbières. − Ça et là dans l’aire du type”; P. pratensis var. humilis sensu Coutinho, Fl. Portugal 1: 104. 1939, non P. pratensis var. humilis (Ehrh. ex Hoffm.) Spenn., Fl. Friburg. 1: 130. 1825, **syn. nov.**] includes plants in which the blade of most basal leaves is 1.2–3.8(–5.5) mm wide, lanceolate or linear-lanceolate, flat or conduplicate, usually delicate, flexible. This variety is distributed in Eurasia, N Africa and Macaronesia (Azores, Madeira and Canary Islands) and is naturalised in N America and Australia. It is widely dispersed on the Iberian Peninsula [**(And.). Spa.**: A Ab Al Av B Bu (C) Cc Co CR Cs (Cu) Ge Gr Gu Hu J L Le Lo Lu M Ma Mu Na O Or P (Po) S Sa Sg So SS (T) Te (To) V Va (Vi) Z Za. **Port**: AAl (BA) (BB) BL DL E Mi TM], where it appears in mountainous areas [650−2400 m a.s.l. (April) May to July (August)].

Finally, ***Poa pratensis*** var. ***angustifolia*** (L.) Sm., Fl. Brit. 1: 105. 1800 [*Poa angustifolia* L., Sp. Pl. 67. 1753, basion., type: “Habitat in Europa ad agrorum versuras” (lectotype designated by Soreng 2000, pg. 254: Herb. Linn. No. 87.12!, excluding second culm from left); *P. brizoides* Vill., Hist. Pl. Dauphiné 2: 126. 1787, nom. illeg., non L. fil., Suppl. Pl. 110. 1782, = *Eragrostis capensis*; *P. villarsii* J.F. Gmel., Syst. Nat., ed. 13, 2: 182. 1791, type: “not expressly indicated”; *P. setacea* Hoffm., Deutschl. Fl., ed. 2, 1: 44. 1800, nom. illeg., non Huds., Fl. Angl. 34. 1762; *P. strigosa* Hoffm., Deutschl. Fl., ed. 2, 1: 44. 1800, type: “In siicioribus elatis; fl. Maj. Iun”; P. pratensis subsp. angustifolia (L.) Lej., Comp. Fl. Belg. 82. 1828; P. angustifolia subsp. brizoides K. Richt., Pl. Eur. 1: 88. 1890; P. pratensis subsp. atlantis Maire, Fl. Afrique N. 3: 101. 1955, type: “Bords des ruisselets, prairies irriguées des collines et montagens siliceuses, rare.- M. Grand Atlas, Mont Gourza vers 2800 m (M.)”], includes plants in which the blade of the basal and shoot leaves is 0.5−1.3 mm wide, linear or setaceous, convolute, usually rigid and brittle. Its distribution area extends across Europe, NW of Africa (Morocco), SW and S Asia and Macaronesia (Canary Islands and Madeira) and it is also introduced in N America. It is scattered over much of the territory covered by *Flora iberica* [**And. Port.**: BL (E) Mi TM. **Spa.**: A Ab Al Av B Bu C Cc Co CR Cu Ge Gr Gu Hu J L Le Lo Lu M Ma Mu Na O Or P PM [Mll] S Sa Sg So (T) Te To V Va Vi Z Za], blooming between April and July, from sea level to 2150 (2400) m.

For a representative list of studied materials, see Suppl. material 1.

#### 2. Poa legionensis

Taxon classificationPlantaePoalesPoaceae

(Laínz) Fern.-Casas & Laínz in Laínz, Contr. Fl. Asturias 83. 1982.

**Figure 2.**
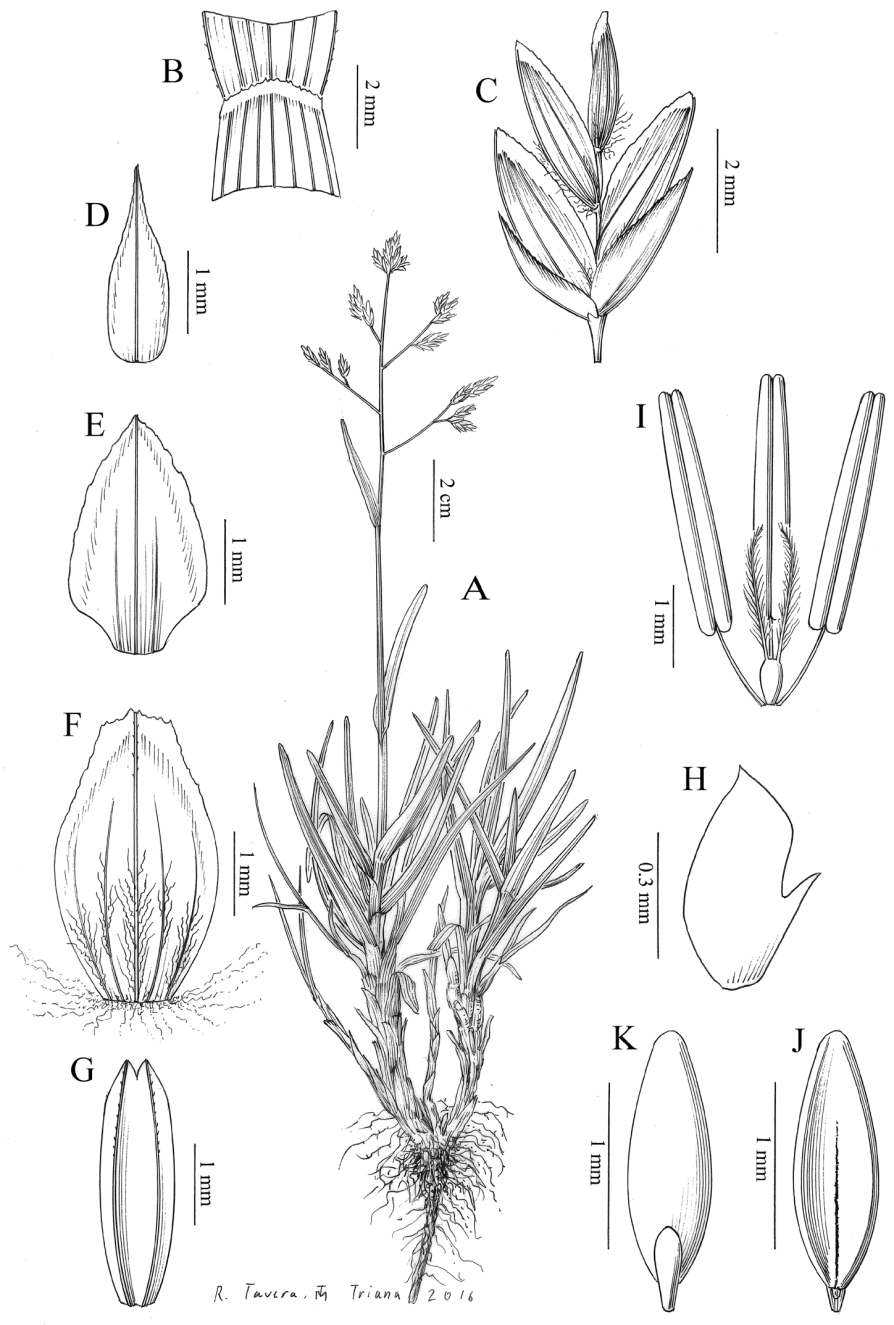
*Poa legionensis* (Laínz) Fern.-Casas & Laínz **A** Habit **B** Detail of the apex of the sheath and ligule, in adaxial view (upper leaf) **C** Spikelet **D** Lower glume, in abaxial view **E** Upper glume, in abaxial view **F** Lemma, in abaxial view **G** Palea, in abaxial view **H** Lodicule **I** Sexual verticils **J** Caryopsis, in adaxial view **K** Caryopsys, in abaxial view. Drawn from MA 314663 and MA 508371.

Poa pratensis var. monticola Merino, Fl. Galicia 3: 337. 1909, **syn. nov.** [Type: “La var. 2.ª cerca de la cumbre de Peña Rubia (Ancares) á unos 1.700 m. s. m.”]. (Type material probably disappeared).
Poa pratensis subsp. legionensis Laínz, Bol. Inst. Estud. Asturianos, Supl. Ci. 15: 43. 1970 [basion.]
Poa alpina subsp. legionensis (Laínz) Rivas Mart. & al., Veg. Alta Mont. Cantábrica 279. 1984.
**Ill.**Ruiz (1991: 31, lam. III); Fig. 2.

##### Type.

“Ut videtur, diffusa per iuga silicea editissima, occidentalia, montium provinciae legionensis (León) et nonnullarum finitimarum. *Holotypus* in herbario meo hispanico boreo-occidentali: iuxta montem Cornón, pr. Lumajo (Villablino, León), ad 1900 m, 1-VII-1959. Insuper specimina legi simillima, per tractum longum satis: in summo Cellón, pr. Arbas (Rodiezmo, León), ad 2000 m, et paulo inferius, ad 1800 m; item, iuxta lacunam celeberrimi Cueto de Arbas, ad 1750 m, supra Leitariegos (Cangas del Narcea, Asturias); denique, paulo infra summam Peña Trevinca, in ditione quidem zamorensi, ad 2075 m.”. [Holotype JBAG-11515-Laínz!].

##### Flowering.

May to July (September).

##### Ecology.

Wet, somewhat nitrified meadows, stony places, swampy areas and psychro-xerophilic pastures (“cervunales”), on granites or slates; 1560−2400 m a.s.l.

##### Distribution.

Endemic to the CN of the Iberian Peninsula. **Spa.**: Av Cc Le Lu O Or (S) Sa (Za). For a representative list of studied materials, see Suppl. material 1.

#### 3. Poa cenisia

Taxon classificationPlantaePoalesPoaceae

All., Auct. Fl. Pedem. 40. 1789.

Poa distichophylla Gaudin, Alpina 3: 39. 1808. [Type: “Sie kommt ziemlich häufig am Ufer der Alpenbäche im Sand vor, .... Auf dem Lioson, auf dem Bonhomme in Savoyen, auf den Walliser Alpen u.s. w. Ṩ. Bl. im Jul. und Aug.”].
Poa pallens Haller fil. ex Gaudin, Alpina 3: 41. 1808. [Type: “Dieses zierliche Gras findet man auf den Weiden und an grasigten Felsen auf den höheren Bergen; auf dem groβen Bernhard; auf Tzermotanaz au-dessus du val de Bagnes; auf dem Bernardin, unweit der Quelle des Hinter-Rheins, u. s. w. Ṩ. Bl. im Jul. un Aug.”].
Brachypodium cenisium (All) P. Beauv., Ess. Agrostol. 101, 155, 174. 1812.
Poa halleridis Roem. & Schult., Syst. Veg. 2: 539. 1817. [Type: “In alpibus Bernensibus. Inn monte Stockhorn. in Valesia”].
Poa cenisia var. halleri Rchb., Icon. Fl. Germ. Helv. 1, ed. 2: 50. 1850. [Type: “In lapidosis rupibusque in Tiroli, Bavaria, Helvetia, Pedemontio”].
Poa cenisia subsp. pallens (Gaudin) Asch. & Graebn., Syn. Mitteleur. Fl. 2(1): 404. 1900.
Poa fontqueri Braun-Blanq., Bull. Soc. Pharm. Montpellier, Comm. SIGMA 87: 220. 1945. [Type: “Hab. In glareosis schistosis vel graniticis regionis subalpinae-alpinea pyrenaeorum orient. satis frequens. Typus: Pic Fontnègre 2650 m.”].
Poa cenisia subsp. fontqueri (Braun-Blanq.) Rivas Mart., Fern. Gonz. & Sánchez Mata, Itinera Geobot. 4: 117. 1990.
Poa cenisia var. fontqueri (Braun-Blanq.) Portal, Poa France Belgique Suisse 91. 2005
**Ill.**Bolòs and Vigo (2001: 384), Portal (2005: 88, 271, sub var. cenisia).

##### Type.

“Secus torrentes sabulosos exsiccatos in monte Cenisio”. (Type material conserved in TO according to Kerguélen 1975, pg. 238).

##### Flowering.

July to September.

##### Ecology.

Grasslands, gravelly areas, rocky slopes and screes on shale, granite or limestone; edaphically indifferent; 1700−2900 m a.s.l.

##### Distribution.

Mountainous areas of C and S Europe. Mountains of N Spain (Pyrenees, Cantabrian Mountains and N Iberian System). **And. Spa.**: Ge Hu L Le Lo (O) P (S) So Z. For a representative list of studied materials, see Suppl. material 1.

#### 4. Poa chaixii

Taxon classificationPlantaePoalesPoaceae

Vill., Fl. Delph. 7. 1785 [1786].

Poa sylvatica Chaix in Vill., Hist. Pl. Dauphiné 2: 128. 1787, nom. illeg., non Poa sylvatica Pollich, Hist. Pl. Palat. 1: 83. 1776.
Poa sudetica Haenke in Jirasek, Beobacht. Reis. Riesengeb. 120. 1791. [Type: “An dem Aupasturze im Thale”].
Poa rubens Moench, Methodus 187. 1794, nom. illeg., non Poa rubens Lam., Tabl. Encycl. 1: 184. 1791; = Eragrostis unioloides).
Poa latifolia Pohl, Tent. Fl. Bohem. 1: 94. 1810, nom. illeg., non Poa latifolia (Osbeck) G. Forst., Fl. Ins. Austr. 8. 1786; = Centotheca lappaceae).
Poa sudetica var. rubens DC. in Lam. & DC., Fl. Franç. éd. 3, 5: 272. 1815. [Type: “Habitat in Haffia inferior”]. Poa sulcata Lag., Gen. Sp. Pl. 3. 1816. [Type: “Legi in sylvis tractus Valgrande dicti, non procul á Pajares oppido”]. Typification supposedly carried out by Fernández Casas & Gamarra (1993: 93), but without establishing an effective lectotypification. Lectotype designated here: “Poa sulcata Lam. / Poa sudetica? / Lagasca iter astur / en Valgrande / Julio.” (label manuscr.): MA 209891].
Cynodon sudeticus (Haenke) Raspail, Ann. Sci. Nat. (Paris) 5: 302. 1825.
Poa chaixii var. rubens (DC.) Asch. & Graebn., Syn. Mitteleur. Fl. 2: 423. 1900.
Poa haemi F. Herm., Bull. Soc. Bot. Bulgar. 3: 43. 1929. [Type: “Habitat: Central-Balkan prope Tulovo Bulgariae, ubi legit lv. Mrkwička Sofia,. (Herbarium regale)”].
**Ill.**Pignatti (1982: 470), Portal (2005: 93, 273).

##### Type.

“In sylvis & pratis alpestribus circa Chaudun prope Vapincum & ad Taillefer”. (Type material probably conserved in GRM, although doubtful according to Kerguélen 1975, pg. 238).

##### Flowering.

June to August.

##### Ecology.

Mountain meadows and grasslands, in brooms, heaths, beeches, oaks and hollies; edaphically indifferent; 1000−2380 (2780) m a.s.l.

##### Distribution.

C and S Europe; naturalised in Finland. N Iberian Peninsula. **And. Spa.**: (Bu) Ge Hu L Le Lo Lu O (Or) P S (So) (Za). For a representative list of studied materials, see Suppl. material 1.

#### 5. Poa glauca Vahl in Oeder, Fl. Dan. 6(17): 3. 1790 subsp. glauca

Taxon classificationPlantaePoalesPoaceae

Poa caesia Sm., Fl. Brit. 1: 103. 1800. [Type: “Ang. Sea-green Meadow-grafs. In Scotiâ. D. Fairbairn. Mountains in Bredalbane. Mr. Mackay”].
Poa nemoralis var. glauca (Vahl) Gaudin, Agrost. Helv. 1: 182. 1811.
Poa nemoralis subsp. glauca (Vahl) Gaudin, Fl. Helv. 1: 240. 1828.
Poa balfourii Parnell, Ann. Mag. Nat. Hist., ser. 1: 10. 1842. [Type: “not expressly indicated”].
Paneion glaucum (Vahl) Lunell, Amer. Midl. Naturalist 4: 222. 1915.
**Ill.**Portal (2005: 98, 278, sub var. glauca).

##### Type.

“Legi tantummodo in paroecia Wang Walders, ad pedes montium, in Finmarkia minus frequens. Prater aliis notis, praesertim colore glau.....”. (Holotype conserved in C according to PAF 2018: Norway: Oppland, Vang, “legi in alpibus Norvegiae Valders versus Vang”, leg. J. Vahl).

##### Flowering.

(July) August to September.

##### Ecology.

Rocky places and forest and scrub grasslands; (1500) 1900−2770 m a.s.l.

##### Distribution.

Circumboreal: Eurasia (extending S to the Pyrenees, S Alps and N of Greece) and Arctic and alpine regions of N America; also Argentina. NE Spain. **(And.). Spa.**: Ge Hu. For a representative list of studied materials, see Suppl. material 1.

#### 6. Poa nemoralis L., Sp. Pl. 69. 1753 subsp. nemoralis

Taxon classificationPlantaePoalesPoaceae

Poa angustifolia var. nemoralis (L.) Huds., Fl. Angl., ed. 2: 41. 1778.
Paneion nemorale (L.) Lunell, Amer. Midl. Naturalist 4: 222. 1915.
**Ill.**Portal (2005: 282).

##### Type.

“Habitat in Europa ad radices montium umbrosas” (lectotype designated by Soreng 2000, pg. 255: icon in Scheuschzer, Agrostogr. Helv. Prodr. t. 2, 1708; epitype designated by Soreng and Edmondson in Soreng 2000, pg. 255: BM).

##### Flowering.

April to August (November).

##### Ecology.

Grasslands in shady, usually deciduous forests and in pastures, margins of alpine wetlands (“borreguiles”) and fissures of rocks; edaphically indifferent; (135) 550−2980 m a.s.l.

##### Distribution.

Europe, temperate Asia and NW Africa (Morocco); introduced in other parts of the world (e.g. Canada, USA, Patagonia and Guatemala). N half and S third of the Iberian Peninsula. **And. Por.**: BA DL TM. **Spa.**: (A) (Ab) Al Av B Bu Ca Cc Cs Cu Ge Gr Gu Hu J L Le Lo Lu M (Ma) Na Or O P S Sa Sg So SS T Te To V Va Vi Z Za.

##### Notes.

*Poa nemoralis* is a polymorphic species with two recognised patterns of variation and numerous transitional forms in the territory encompassed by *Flora iberica*. The first recognised variety, ***Poa nemoralis*** var. ***nemoralis*** [*Poa cinerea* Vill., Hist. Pl. Dauphiné 2: 126. 1787, type: “Il vient au même endroit que le précédent -*Poa angustifólia*-.”; *P. debilis* Thuill., Fl. Env. Paris ed. 2: 43. 1799, type: “Habitat in pratis”; *P. miliacea* DC. in Lam. & DC., Fl. France ed. 3, 3: 64. 1805, type: “ .. par M. Ramond, qui l’a trouvée dans les Pyrénées; ...”; P. nemoralis var. montana Gaudin, Alpina 3: 27. 1808, type: “In den Wäldern des Jura Ṩ. Bl. im Jun. un Jul.”; P. nemoralis subsp. vulgaris Gaudin, Agrost. Helv. 1: 179. 1811, nom. superfl.; P. nemoralis subsp. firmula Gaudin, Agrost. Helv. 1: 181. 1811, type: “In plantici dumetis non rara”; P. nemoralis subsp. coarctata Gaudin, Agrost. Helv. 1: 185. 1811, type: “In aridis apicisque, ad muros etiam alpinis hawd infrequens”; P. nemoralis var. miliacea (DC.) Godr. in Gren. & Godr., Fl. France 3: 541. 1856; P. nemoralis var. eunemoralis Hack. in Briq., Prodr. Fl. Corse 1: 141. 1910, nom. inval.], is widely distributed in the area covered by *Flora iberica* [**And. Por.**: BA DL TM. **Spa.**: Al Av B Bu Ca Cc Cs Cu Ge Gr Gu Hu J L Le Lo Lu M Ma Na Or O P S Sa Sg So SS T Te To V Va Vi Z Za]. This taxon includes plants that are usually green or sometimes glaucous, generally with smooth and flexible stems and with most leaves flat and flexible, linear or linear-lanceolate and erect or erect-patent. The other variety, ***Poa nemoralis*** var. ***rigidula*** Mert. & W.D.J. Koch, Deutschl. Fl. 1: 617. 1823 [type: “Auf Wiesen, im Gebüsche der Triften, am Saume der Wälder”], is dispersed throughout the region [**And. Port.**: TM. **Spa.**: (A) (Ab) Al Av Bu Cc Gr Hu L M Na P S Sg Te Z] and includes plants that are usually glaucous, with rigid and often rough stems and usually convolute leaves that are setaceous, linear or linear-lanceolate, ± rigid and erect. For a representative list of studied materials, see Suppl. material 1.

#### 7. Poa compressa

Taxon classificationPlantaePoalesPoaceae

L., Sp. Pl. 69. 1753.

Poa planiculmis Weber, Suppl. Fl. Holsat. 3. 1787. [Type: “Habitat in collibus prope Neumuhlen”].
Poa compressa var. depauperata Mutel, Fl. Franç. 4: 81. 1837. [Type: “plante étiolée, forêts des Alpes et du Jura”].
Poa compressa var. langeana (Rchb.) W.D.J. Koch, Syn. Fl. Germ. Helv. ed. 2: 932. 1844.
Paneion compressum (L.) Lunell, Amer. Midl. Naturalist 4: 222. 1915.
Poa cenisia subsp. sardoa E. Schmid, Vierteljahrsschr. Naturf. Ges. Zürich 78: 239. 1933. [Type: “Perdas Crapias am Gennargentu, 1820 m, Gneissfelsflur (22.VII.1923, b.) ...”].
**Ill.**Portal (2005: 95, 276), Soreng and Peterson (2012: 11, fig. I, F-M).

##### Type.

“Habitat in Europae & Americae septentrionalis siccis, muris, tedis” (lectotype designated by Soreng 2000, pg. 255: LINN-87.41!).

##### Flowering.

May to September (October).

##### Ecology.

Meadows and grasslands, forests, gravelly areas, margins of roads and slopes; edaphically indifferent, although preferring basic substrates; (75) 540−1990 (2300) m a.s.l.

##### Distribution.

Circumboreal (most of Europe, to SW Asia); introduced in N, C and S America (Peru, Argentina) and Australia. CN and E Spain, rarer in the south. **And. Port.**: TM. **Spa.**: A (Ab) B (Bi) Bu (Cs) Cu Ge Gr Gu H Hu J L (Le) Lo (Lu) M Na (Or) P S Sa Sg So T Te (V) Va Vi (Z) Za. For a representative list of studied materials, see Suppl. material 1.

##### Notes.

Plants of this species usually have a glaucous green colour. The lower (and sometimes higher) leaves of many studied herbarium specimens are missing their blades and are frequently fragmented with the ligule exposed. Inflorescences of *P. compressa* are frequently narrow and interrupted, with almost adpressed branches and spikelets are variable in size and number of flowers. The spikelets may be long, almost always entirely glabrous and glaucous, with 4–9 flowers or short and bear 2–5 flowers, and this variability may be present in the same population or even on the same plant. *Poa compressa* has not been previously listed in the flora of Portugal.

#### 8. Poa laxa Haenke in J. Jirasek, Beobacht. Reis. Riesengeb. 118. 1791 subsp. laxa

Taxon classificationPlantaePoalesPoaceae

Poa laxa ß *pallida* Lange, nom. nud., in sched. (C 10022611; COI-Willk. 36552), **syn. nov.**
**Ill.**Bolòs and Vigo (2001: 386), Portal (2005: 108, 281).

##### Type.

“[Haenke 1791: 116, Schneekoppe] der kahle, steinigte Gipfel” [= the summit area of Mt Sněžka, NE Bohemia, at the Polish border]” (lectotype designated by Kirschner et al. 2007, pg. 349: *Poa laxa* a me descripta in Actis Societ. Boh. Anno 1787. *Poa* Halleri historia Nr. 1457. Lecta in Sudetis et in Styriae Alpibus, T. Haenke (PR); epitype designated by Kirschner et al. 2007, pg. 349: Bohemia, the Krkonoše Mts, Mt Sněžka, scree site just below the summit plateau at the beginning of the track called Jubilejní cesta, 50°44'10"N, 15°44'25"E, 3 Jul 2007, J. Zahradníková&L. Harčariková s.n: PRA 349; isoepitypes: PR, PRC).

##### Flowering.

July to September.

##### Ecology.

Rocky places, stony places and high mountain waterfalls, on shale, schist and granite; (1900) 2300−3150 m a.s.l.

##### Distribution.

C and N of Europe, reaching the Carpathians, Balkans, Apennines and Pyrenees, and N America. N and NE Iberian Peninsula. **And. Spa.**: Ge Hu L (P). For a representative list of studied materials, see Suppl. material 1.

#### 9. Poa minor

Taxon classificationPlantaePoalesPoaceae

Gaudin, Alpina 3: 44. 1808.

Poa laxa subsp. minor (Haenke) Hooker fil., Stud. Fl. Brit. Isl., ed. 1: 444. 1870.
Poa laxa var. minor (Haenke) Fiori in Fiori & Paoletti, Fl. Italia 1: 86. 1898.
**Ill.**Bolòs and Vigo (2001: 387), Portal (2005: 111, 281).

##### Type.

“Dieses schöne Gras findet man wie das vorige, auf hohen Gebirgen; auf dem Bernhard, auf den Bergen oberhalb Ber und Aigle u. s. w. Bl. im Jul. und Aug.”. (Type material conserved in LAU according to Kerguélen 1975, pg. 241).

##### Flowering.

(July) August to September.

##### Ecology.

Talus slopes, stony places and fissures of rocks, wet and sheltered grasslands, on limestone and schist; 2000−3350 m a.s.l.

##### Distribution.

Mountains of S Europe: Sierra Nevada, Pyrenees, Alps, Balkans and Carpathians. N and SE Spain: Cantabrian Mountains, Pyrenees and Sierra Nevada. **(And.). Spa.**: Ge Gr Hu (Na) O S.

##### Two subspecies of *P. minor* are recognised in the territory encompassed by *Flora iberica*. A key to their identification is given below:

**Table:** 

1	Blades of most leaves conduplicate, rarely flat, those of basal and shoot leaves 0.4–1.6(–2.1) mm wide; branches of the inflorescence flexuous; spikelets ovate-oblong; lemma hairy at the nerves and the base, hairs of the latter usually longer than the width of the lemma; anthers 0.8–1.1 mm	**a. subsp. minor**
−	Blades of most leaves flat, rarely conduplicate, those of basal ones 1.5–2.3 mm wide; branches of the inflorescence straight; spikelets oblong; lemma glabrous or only weakly hairy at the nerves; anthers (1–)1.2–1.7 mm	**b. subsp. nevadensis**

**a. subsp. minor**

*Poa supina* Panz. in Sturm, Deutschl. Fl. 34: 1. 1812, nom. illeg., non *Poa supina* Schrader, Fl. Germ. 289. 1806.

*Poa pyrenaica* Lange ex Willk. in Willk. & Lange, Prodr. Fl. Hispan. 1: 80. 1861, nom. inval., pro syn. 

Poa jacetana gr. laxa P. Montserrat, nom. nud., in sched. (MA 291693), **syn. nov.**

**Flowering.**

(July) August to September.

**Ecology.**

Talus slopes, stony places and fissures of limestone rocks, wet and sheltered high mountain locations; rarely on schist; 2000−3207 m a.s.l.

**Distribution.**

Mountain systems of S Europe (Cantabrian Mountains, Pyrenees, Alps, Balkans and Carpathians). N Spain: Cantabrian Mountains and Pyrenees. **(And.). Spa.**: Ge Hu L Na O S. For a representative list of studied materials, see Suppl. material 1.

**b. subsp. nevadensis Nannf. in Font Quer, Exsicc. Fl. Iber. Select. Cent. 3: n. 201. 1935.**

*Poa laxa* sensu Boiss., Voy. Bot. Espagne 659. 1842, non *Poa laxa* Haenke in J. Jirasek et al., Beobacht. Reis. Riesengeb. 118. 1791.

Poa minor var. nevadensis (Nannf.) Á.M. Hern., Acta Bot. Malac. 2: 35. 1976, comb. inval., **syn. nov.**

**Ill.** Fig. 3.

**Type.**

“Baetica: in schistosis montium Sierra Nevada, 1. Cerro de la Alcazaba dicto, ad 3000 m alt., Leg. Font Quer, 28 aug. 1923” (lectotype designated here: “Institut Botanicum Barcinonense / Flora Iberica Selecta / Cent. III Dec. 1935 / 201. Poa minor Gaud. / Fl. Helvet., I, p. 253 (1828). / ssp. nevadensis Nannf. nov. ssp. / Poa laxa Boiss., Voy. Bot. Esp., II, p. 659, non Haenke. / Baetica: in schistosis montium Sierra Nevada, l. Cerro de la Alca- / zaba dicto, ad 3000 m alt. Cotypus. / Leg. Font Quer, 28 aug. 1923. / Obs.: Differt a typo foliis tenuioribus, planis; ligulis brevioribus / (2 mm non excedentibus); spiculis angustioribus et longioribus; / floribus distantioribus; glumis valde inaequelibus; gl. I 2-2,5 mm / longis, gl. II 2,2-3,= mm; antheris paulo longioribus et angustiori-/ bus (longit. 1,1-1,3 mm). J. A. Nannfeldt.” (label printed): UPS-V-873177, top specimen on the right; isolectotypes designated here: BC 990150, BC 87706, BC 87707; GDA 31029, 31030, 31031; MA 11385; MAF 28595).

**Flowering.**

July to September.

**Ecology.**

Pastures and wet stony places in high mountain locations, on schist; (2500) 3000−3350 m a.s.l.

**Distribution.**

Endemic to SE Spain: Sierra Nevada. **Spa.**: Gr. For a representative list of studied materials, see Suppl. material 1.

**Figure 3.**
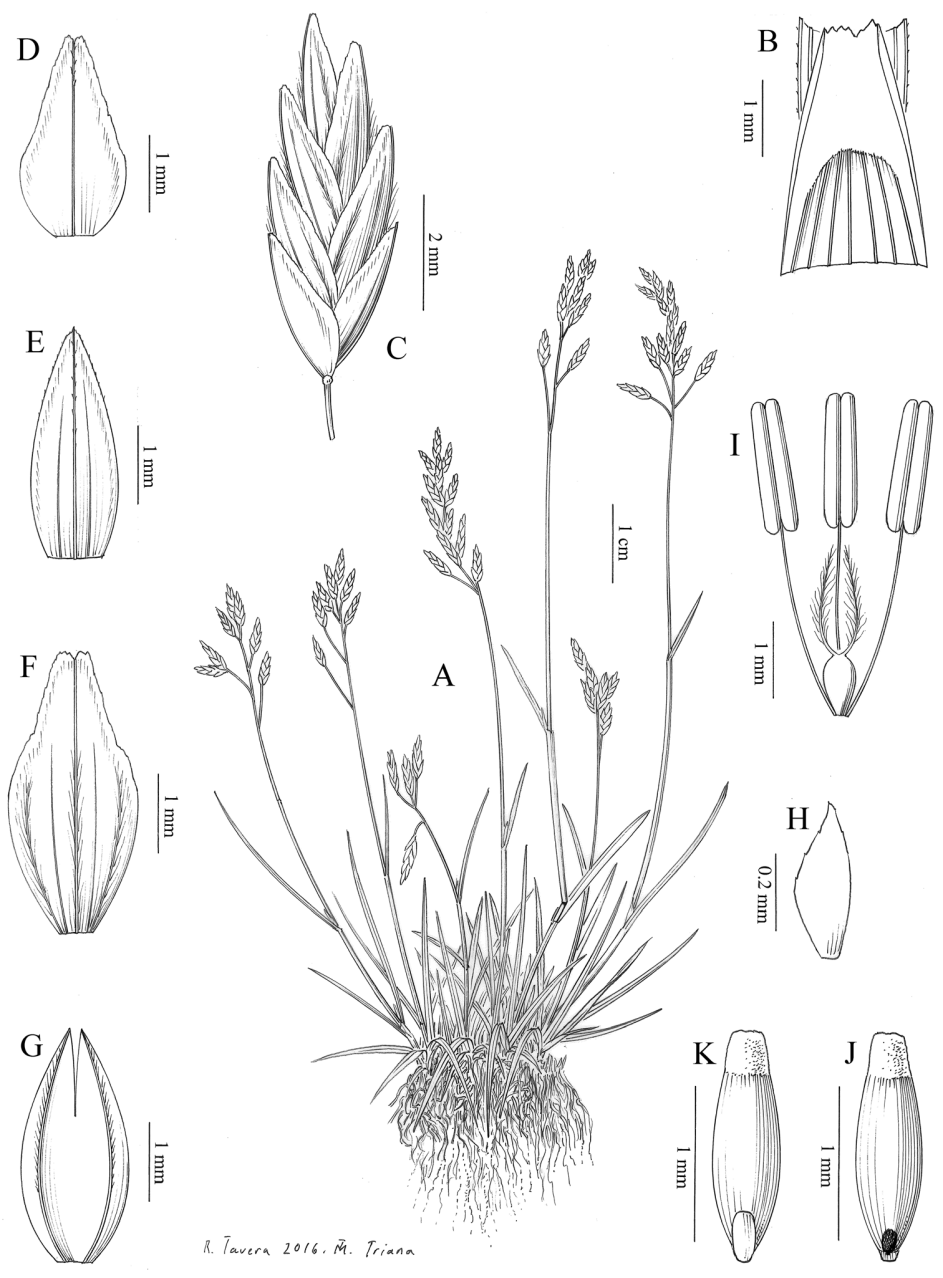
Poa minor subsp. nevadensis Nannf. **A** Habit **B** Detail of the apex of the sheath and ligule, in adaxial view (upper leaf) **C** Spikelet **D** Lower glume, in abaxial view **E** Upper glume, in abaxial view **F** Lemma, in abaxial view **G** Palea, in abaxial view **H** Lodicule **I** Sexual verticils **J** Immature caryopsis, in adaxial view **K** Caryopsys, in abaxial view. Drawn from GDA 31029 and MA 422687.

#### 10. Poa trivialis L., Sp. Pl. 67. 1753 subsp. trivialis

Taxon classificationPlantaePoalesPoaceae

Poa dubia Leers, Fl. Herborn. 28, tab. 6, fig. 5. 1775. [Type: “H. in pratis humidiusculis ubique frequens; ad fossas; an der Dillae & Mühlbach copiose; etiam fisscioribus locis gramineis pervulvaris”].
Poa scabra Ehrh., Beitr. Naturk. 6: 83. 1791, nom. inval.
Poa stolonifera Haller ex Muhl., Descr. Gram. 139. 1817, nom. illeg., non Poa stolonifera Bellardi, Mem. Reale Accad. Sci. Torino 5: 215. 1792.
Poa trivialis α vulgaris Rchb., Icon. Fl. Germ. Helv. 1: 37, fig. 1653, 1654. 1834, nom. superfl.
Poa feratiana Boiss. & Reut., Pugill. Pl. Afr. Bor. Hispan. 128. 1852. [Type: “Hab. In sylvâ Irati Pyrenaeorum occidentalium loco Erreca-Idorra (Férat in herb. Fauché!) Fl. Julio”] (lectotype designated by Burdet et al. 1981, pg. 577: specimen indicated with arrows, G00176550!).
Poa sylvicola Guss., Enum. Pl. Inarim.: 371, tab. 18. 1854. [Type: “In sylvaticis apricis ubique vulgatissima; nec non prope Neapolina, et Stabias”].
Poa trivialis var. umbrosa Balansa, Bull. Soc. Bot. France 21: 16. 1874. [Type: “Rhizè, dans les foréts, les lieux humides”].
Poa trivialis var. sylvicola (Guss.) Hack., Verh. K. K. Zool.-Bot. Ges. Wien 1890: 127. 1890.
Poa attica sensu Pérez Lara, Anales Soc. Esp. Hist. Nat. 15: 406 (1886), non Poa attica-Boiss. & Heldr. in Boiss., Diagn. Pl. Orient. ser. 1, 13: 57 (1854).
Poa attica var. gaditana Pérez Lara ex Willk., Suppl. Prodr. Fl. Hispan. 22. 1893, **syn. nov.** [Type: « …provinciae Gadit. Haud frequens (pr. Jerez in loco et Albaladejo et in Dehesa del Torongil; pr. Grazalema ad Huertas de Benamahona, PER. LARA!) »].
Poa trivialis var. flaccida Willk. ex J.J. Rodr., Fl. Menorca 152. 1904, **syn. nov.** [Type: “Barranco de Son Blanc en sitios frescos”] (lectotype designated here: first label: “Glumelle glabra. Ax. de l’epilles numi / à la base des glumelles inferieures d’un “ faisceau de poile longe et soyeux. / Gaines sublisses. -illegible- noueux à / leur base, lisses au sommet. / = P. attica Boiss. Heldr. var. flaccida.” (manuscr.); second label: “J. J. Rodriguez. (printed) Plantas de Menorca. (Baleares.) (printed) / Poa trivialis L.? (manuscr.) / var. (¿) flaccida Wk. ined (manuscr.) / Localidad (printed) Barranco de Son Blanc (Algar) (manuscr.) / Estacion (printed) Sitios frescos (manuscr.) / Epoca (printed) 28 mayo 1874 (manuscr.) / Leg. Rodríguez (impreso)”: COI-Willk. 36527; isolectotype designated here: HGM 3121-1).
Poa trivialis subsp. sylvicola (Guss.) H. Lindb. fil., Oefvers. Förh. Finska Vetensk.-Soc. 38(13): 9. 1906.
Poa trivialis “rasse” majorica [*majorcica*] F. Hermann, Verh. Bot. Vereins Prov. Brandenburg 54: 252. 1914, **syn. nov.** [Type: “An Rainen und Wegrändern sammelte …”].
Poa trivialis f. majorica (F. Hermann) Knoche, Fl. Balear. 1: 310. 1921, **syn. nov.**
Poa trivialis f. flaccida (Willk. ex J.J. Rodr.) Knoche, Fl. Balear. 1: 311. 1921, **syn. nov.**
Poa trivialis var. obtusata Maire, Fl. Afr. N. 3: 99. 1955, nom. inval.
Poa trivialis subsp. feratiana (Boiss. & Reut.) Á.M. Hern., Acta Bot. Malac. 2: 33. 1976.
Poa portalii H. Scholz, Willdenowia 42: 293 (2012), **syn. nov**. [Type: Holotype: France: Aquitaine, Pyrenees Atlantiques, Iraty, c. 50 m behind camping place on a wet depression at border of a foot path in woodland, 1150 m, 11.8.2010, Böhling 15255 (B, as “Poa supina”)]. Holotype B 100558216 (seen by Soreng in 2015, pers. comm.).
Poa trivialis var. modesta Caball., nom. nud., in sched. (MA 11644, MA 11627), **syn. nov.**
Poa trivialis f. biflora Bernis, Flora Maragata, nom. nud., in sched. (MA 11615; MA 11617), **syn. nov.**
Poa verticillata auct., non L. (MA 11631).
Poa trivialis var. contracta Pérez Lara, nom. nud., in sched. (MAF 28658, MAF 28569, MAF 28655), **syn. nov.**
**Ill.**Ruiz (1991: 29, lam. II), Devesa (1987: 263, sub P. trivialis subsp. trivialis and subsp. sylvicola).

##### Type.

“Habitat in Europae pasenis” (neotype designated by Soreng 2000, pg. 256: Hudson 16, Herb. Linn. No. 87.9!).

##### Flowering.

(March) April to July.

##### Ecology.

Pastures, hygrophilous grasslands (stream edges, peat bogs, reed patches and meadows) and very wet soils of deciduous forests (e.g. alders, ashes, chestnuts and oaks); edaphically indifferent; 0−2000 (2150) m a.s.l.

##### Distribution.

Europe, N Africa, Asia and Macaronesia (Azores, Madeira and Canary Islands); introduced in other parts of the world. Most of the Iberian Peninsula and Balearic Islands. **And. Port**: AAl Ag BA BB BL (BAl) DL E Mi R TM. **Spa.**: A Ab Al Av B Ba Bu C Ca Cc Co CR Cs Ge Gr Gu H Hu J L Le Lo Lu M Ma (Mu) Na O (Or) P PM[Mll Mn] Po S Sa Se Sg So (SS) (T) (Te) To V Va Vi Za (Z). For a representative list of studied materials, see Suppl. material 1.

##### Notes.

*Poa trivialis* is variable with regard to habit, leaf size and inflorescence morphology. The most distinctive feature of this species is the elongated ligule, which is always longer than the width of the leaf blade, ovate or ± triangular in the basal leaves and irregularly dentate or bilobed with an acute apex in the uppermost ones. In addition, the spikelets have 2 or 3 flowers and the sharp, arched glumes converge around the lemma. The base of the lemma is very woolly or extremely rarely glabrous and the hairs are clearly longer than its width.

Some plants have somewhat thickened and constricted stolons, with a more or less moniliform appearance. These individuals were described as *Poa sylvicola* Guss. (= P. attica var. gaditana Pérez Lara ex Willk.; = P. trivialis var. umbrosa Balansa). According to Soreng (pers. comm.), P. trivialis subsp. sylvicola (Guss.) H. Lindb. fil. is common in the Mediterranean region, while subsp. trivialis is rather infrequent and, conversely, subsp. sylvicola is infrequent northwards. Other characteristics of subsp. sylvicola are smoother sheaths and the consistent presence of hairs on the lower part of the marginal lemma veins vs. their absence in subsp. trivialis. Practically speaking, the marginal vein is hairy in Mediterranean populations but glabrous or nearly so in northern ones. In both types of populations, however, the differing combinations of forms of these characters makes it almost impossible to delimit these two taxa. Consequently, we have opted not to recognise them as separate subspecies.

*Poa feratiana* Boiss. & Reut. is also included here as a synonym of *P. trivialis*. Plants labelled as *P. feratiana* on herbarium sheets had 2 flowers per spikelet, which is diagnostic for this species, but this characteristic is also very common on most studied sheets of *P. trivialis*. In addition—as indicated by Hernández Cardona (1976) after studying the type material (G-herbarium Boissier)—some of the characteristics attributed to this species in the original description were incorrect. For instance, the number of veins of the lemma is actually 5, not 3, a feature likely overlooked by the original authors because the marginal veins are usually very close to the edge. As another example, the lemma is indeed woolly at the base, as is common in *P. trivialis*.

Plants with some of their spikelets completely sterile and reduced to a set of whitish or hyaline membranes are also known.

#### 11. Poa flaccidula

Taxon classificationPlantaePoalesPoaceae

Boiss. & Reut., Pugill. Pl. Afr. Bor. Hispan. 128. 1852.

Poa balearica Porta, Nuov. Giorn. Bot. Ital. 19: 324. 1887. [Type: “M. Ad pedes rupium praeruptarum m. Coma den Ar-bona” (lectotype designated by Rosselló and Sáez 2000, pg. 141, second on left specimen: G].
Poa trivialis subsp. balearica (Porta) Gand., Nov. Consp. Fl. Eur. 506. 1910, **syn. nov.**
Poa trivialis f. balearica (Porta) Knoche, Fl. Balear. 310. 1921.
Poa trivialis var. balearica (Porta) O. Bolòs & Molinier, Collect. Bot. (Barcelona) 5: tab. 7. 1958, comb. inval., **syn. nov.**
Poa zapateri Gandoger, nom. in sched. (MA 11500). [Type: “Sierra de Albarracín”]
Poa ventalloi Sennen, nom. nud., in sched. (Sennen BC-966297).
**Ill.**Devesa (1987: 264).

##### Type.

“Habitat in umbrosis septentrionalibus jugi Cerro de San Cristoval et Sierra de la Nieve ditionis Serrania de Ronda Junio 1849 (Boiss. et Reuter)” (lectotype designated by Hernández Cardona 1978, pg. 104-105, 337, left specimen on the sheet from Cerro de San Cristóbal, Reuter, 6-1849: G 00176652!).

##### Flowering.

April to July.

##### Ecology.

Stony places, cliffs, scrub clearings and understoreys, on limestone; (200) 700−2030 m a.s.l.

##### Distribution.

W Mediterranean region (S France [Provence], peninsular Spain, Balearic Islands and NW Africa [Morocco and Algeria]). S and E half of peninsular Spain and Mallorca. **And. Spa.**: A (Ab) (Al) B Bu Ca (Co) CR Cs Cu Gr Gu Hu J L Ma (Mu) (Na) PM[Mll] (S) So (T) Te To V Z. For a representative list of studied materials, see Suppl. material 1.

##### Notes.

Spikelets in this species usually have 2–3 flowers, but can have 3–7, a rare phenomenon observed more frequently in populations in NE Spain. The most distinctive characteristic of *Poa flaccidula* is the sericeous or appressed-hairy indumentum of the intervein zone of both the lemma and palea. This species is sometimes confused with *P. annua*, but, along with other differences, the latter is an annual, not a perennial. *Poa flaccidula* can also be confused with *P. trivialis*, which, like *P. flaccidula*, has a ligule that is longer than the width of the leaf blade and possesses hairs at the base of the lemma that are longer than its width; however, both the palea and the internerval surface of the lemma is glabrous in *P. trivialis*.

P. flaccidula subsp. guadianensis F.M. Vázquez, Folia Bot. Extremad. 9: 66 (2016) has recently been described from Extremadura (SW Spain), but examination of the type material (HSS 65616; COF 62937 isotypus) reveals that this taxon is in no way attributable to *P. flaccidula*. The most we can say, given the immaturity of the specimens, is that it may be of hybrid origin, with *P. bulbosa* possibly one of the parents.

#### 12. Poa annua L., Sp. Pl. 68. 1753 subsp. annua

Taxon classificationPlantaePoalesPoaceae

Poa annua var. viridis Lej. & Courtois, Comp. Fl. Belg. 1: 80. 1828. [Type: “not expressly indicated”].
Poa ovalis Tineo, Pl. Rar. Sicil., fasc. 2: 21. 1846. [Type: “In pascuis montosis, apricis, palustribus. Cotrano al Gurgo lo Drago”].
Poa annua var. aquatica Asch., Fl. Brandenburg 1: 844. 1864. [Type: “Provinz Brandenburg S. Altd. Sumpf hinter dem Pfarrgarten”].
Poa annua var. typica Beck, Fl. Nieder-Österreich: 84. 1890, nom, inval.
Poa annua var. ovalis (Tineo) Trab. in Batt. & Trab., Fl. Algérie (Monocot.) 2: 206. 1895.
Poa annua f. plicata prostrata Sennen, Pl. Espagne n. 605. 1908, nom. nud., in sched. (MA 11165), p.p., **syn. nov.**
Poa annua var. lanuginosa Sennen, Diagn. Nouv. sér. 1933: 209, n. 8980. 1936. [Type: “Hab.- Maroc: Melilla à Rostrogordo. Leg. Hno. Mauricio”] (lectotype designated here: “1933.−Plantes d’Espagne. −F. Sennen / N° 8980 / Poa annua L. / var. lanuginosa Sennen / Maroc: Melilla, à Rostrogordo / 2−III Leg. Hno. MAURICIO” (label printed): specimen upper on the left, BC 119353; isolectotype: MA 11155).
Poa annua f. lanuginosa Sennen & Mauricio, Cat. Fl. Rif Orient.: 132. 1934, nom. nud., **syn. nov.**
Poa annua var. pilantha Ronniger, Verh. Deutsch. Bot. Ges. Wien 88-89: 97. 1941. [Type: “not expressly indicated”, but the material was collected on the island of Zante, Ionian Islands, Greece].
P. annua subsp. pilantha (Ronniger) H. Scholz, Ber. Deutsch. Bot. Gesell. 81: 19. 1968.
Ochlopoa annua (L.) H. Scholz, Ber. Inst. Lanschafts- Pflanzenökologie Univ. Hohenheim, Beih. 16: 58. 2003.
Ochlopoa annua subsp. pilantha (Ronninger) H. Scholz & Valdés, Willdenowia 36: 661. 2006.
**Ill.**Ruiz (1991: 27, lam. I), Soreng and Peterson (2012: 14, fig. 2A–E), Devesa (1987: 261).

##### Type.

“Habitat in Europa ad vias” (lectotype designated by Soreng 2000, pg. 254: right-hand plant, Herb. LINN No. 87.17!).

##### Flowering.

All year.

##### Ecology.

Pastures and grasslands along roads, fallow fields, gardens, margins of watercourses and more or less nitrified soils of all types; edaphically indifferent; 0−2100 m a.s.l.

##### Distribution.

Cosmopolitan, although apparently of Mediterranean origin. Throughout the Iberian Peninsula and Balearic Islands. **And. Port**: AAl Ag BA BAl BB BL DL E Mi (R) TM. **Spa.**: A Al Av B Ba Bi Bu C Ca Cc Co CR Cs Cu Ge Gr Gu H Hu J L Le Lo Lu M Ma Mu Na O (Or) P PM [Mll, Mn] Po S Sa Se Sg So SS T Te To V Va Vi Z Za. For a representative list of studied materials, see Suppl. material 1.

##### Notes.

Plants are found in the territory covered by *Flora Iberica* that have hairy lemmas, at least towards the internerval basal zone, with this indumentum being more perceptible in apical flowers of the spikelet. This characteristic is usually accompanied by a very dense silky indumentum in the veins. In other cases, the spikelet has lemmas with a glabrous internerval surface, usually accompanied by a lower density of indumentum in the nerves, with sometimes even the medium ones being glabrous or glabrescent. The first variation corresponds to Poa annua var. lanuginosa Sennen (Diagn. Nouv. sér. 1933: 209, n. 8980. 1936), a name that prevails over the name P. annua var. pilantha Ronninger (Verh. Deutsch. Bot. Ges. Wien 88-89: 97. 1941). When Scholz in Ber. Deutsch. Bot. Gesell. 81: 19 (1968) raised Ronninger’s taxon to the subspecies category, he stated that the distribution of this subspecies was Mediterranean (e.g. Greece, Italy, Spain and Morocco) and extra-Mediterranean for the type subspecies. Although plants of Mediterranean environments in the Iberian Peninsula tend to have hairier lemmas, we have also found specimens assignable to var. annua and, conversely, we have observed plants with hairy lemmas in typically Eurosiberian areas (e.g. Lugo, Minho, Oviedo and Santander) and even Macaronesia (e.g. Madeira).

In certain populations, some spikelets are completely sterile and reduced to a set of whitish or hyaline membranes. Although infrequent, plants with loosely antrorse-scabrid inflorescence branches have been detected, perhaps as a result of hybridisation with other species (e.g. MA 420475, MA 449625).

DNA sequence data support the hypothesis that *P. annua*, a tetraploid species, has arisen by hybridisation—and subsequent polyploidisation—between two Eurasian diploid species, the annual *P. infirma* Kunth and the rhizomatous perennial *P. supina* Schrad. (Soreng et al. 2010; Mao and Huff 2012), as suggested by Nannfeldt (1937).

#### 13. Poa infirma

Taxon classificationPlantaePoalesPoaceae

Kunth in Humb., Bonpl. & Kuntz, Nov. Gen. Sp. 1: 158. 1816.

Megastachya infirma (Kunth) Roem. & Schult., Syst. Veg. 2: 585. 1817.
Eragrostis infirma (Kunth) Steud., Nomencl. Bot. ed. 2, 1: 563. 1840.
Poa annua var. exilis Tomm. ex Freyn, Verh. K. K. Zool.-Bot. Ges. Wien 27: 469. 1878. [Type: “So auf sonnigen, trockenen Grasplätzen der Macchien, meist in Gesellschaft von Asterolinon, Euphorbia peploides, E. xigua und anderen Zwergpflanzen stellenweise häufig, bisher aber nur läng der Küste von Fasana bis Medolino; auch auf S. Marina (Tommasini 1872)”].
Poa annua var. remotiflora Hack. ex Batt. & Trab., Fl. Algérie Monocot. 206. 1895. [Type: “Lieux humides et région montagneuse, Rouïba, Teniet-el-Haàd”].
Poa annua subsp. exilis (Tomm. ex Freyn) Murb. in Asch. & Graebn., Syn. Mitteleur. Fl. 2: 389. 1900.
Poa remotiflora (Hack. ex Batt. & Trab.) Murb., Acta Univ. Lund. 36 Afd. 2. n. 1: 22. 1900, nom. illeg., non Poa remotiflora Rupr., Fl. Samojed. Cisural. 63. 1845.
Poa exilis (Tomm. ex Freyn) Murb. ex Nannf., Acta Univ. Lund., ser. 2, 1(4): 73. 1906.
Poa annua f. plicata prostrata Sennen, Pl. Espagne n. 605. 1908, nom. nud., in sched. (MA 11165), p.p., **syn. nov.**
Poa annua var. plicata Sennen, Pl. d’Espagne w.n. 1908, nom. nud., in sched. (MA 11142).
Poa annua var. laxiflora Sennen, Pl. d’Espagne n. 606. 1908, nom. nud., in sched. (MA 11143).
Poa annua subsp. remotiflora (Hack. ex Batt. & Trab.) Jansen & Wachter, Fl. Nederl. 1(2): 78. 1951, nom. illeg.
Poa annua L. var. spiciformis Palau Ferrer, Pl. Baleares n. 793. 1955, nom. in sched. (GDA 30993; MA 168790), **syn. nov.**
Ochlopoa infirma (Kunth) H. Scholz, Ber. Inst. Lanschafts-Pflanzenökologie Univ. Hohenheim Beih. 16: 59. 2003.
**Ill.**Ruiz (1991: 27, lam. I), Soreng & Peterson (2012: 14, fig. 2F–H), Devesa (1987: 262).

##### Type.

“Crescit in frigidis regni Novogranatensis, inter Fontibon, Suba et Santa Fe de Bogota, alt. 1360 hexap. Floret Ausgusto”. [Holotypus P-HUMB; isotypus: B-WILLD-1974 pl. 223, LE-TRIN-2638.01 fragm. & illustr., US-1851276 fragm. ex P, US-2851277 fragm. ex P-HUMB) (designated by Soreng and Peterson 2012, pg. 43)].

##### Flowering.

October to May (July).

##### Ecology.

Therophytic pastures and ruderal places, preferably in sandy soils; edaphically indifferent; 2−1000 m a.s.l.

##### Distribution.

W Europe, Mediterranean, Macaronesian and Irano-Turanian regions extending to India; introduced in Australia and the Americas. Scattered across the Iberian Peninsula and Balearic Islands. **Port**: AAl Ag BA BAl E Mi (R) TM. **Spa.**: A (Ab) Al Av B Ba (C) Ca Cc Co CR Ge Gr H Hu J (L) Lo M Ma (Mu) O (Or) (Po) PM [Mll Mn] S Sa Se Sg Te To V Va (Z) Za. For a representative list of studied materials, see Suppl. material 1.

##### Notes.

This species is clearly differentiated from the previous one, not only by the small size of its anthers, but also by the smaller size of the leaves, which, in most cases, are not more than 2 mm wide and have margins that are barely scabrid or even smooth. Most populations comprise plants of small size (up to 22 cm).

Plants with some spikelets completely sterile and rudimentary, whitish or hyaline have been detected in some populations (e.g. province of Granada, GDA 15557).

#### 14. Poa supina

Taxon classificationPlantaePoalesPoaceae

Schrad., Fl. Germ. 1: 289. 1806.

Poa annua var. varia Gaudin, Alpina 3: 29. 1808. [Type: “An den Bächen, auf den höheren Apen; auf der Scheideck. Seringe. Auf dem Gotthard häufig. Ø. Bl. im Sommer”].
Poa annua subsp. varia (Gaudin) Gaudin, Agrost. Helv. 1: 189. 1811.
Poa annua var. supina (Schrad.) Spenn., Fl. Friburg. 1: 127. 1825.
Poa annua subsp. supina (Schrad.) Husn., Graminées 51. 1898.
Poa exigua Foucaud & Mandon ex Husn., Gram. Fr. Belg. 88. 1899, nom. illeg., non Poa exigua Dumort., Gramin. Belg. 113. 1824.
Poa annua f. macranthera Lit. & Maire in Jahand. & Maire, Cat. Pl. Maroc 1: 66. 1931. [Type: “Sierra Nevada. Pyrénées. Corse. Alpes.”] .
Poa supina f. exigua Gamisans, Candollea 29: 48. 1974. [Type: “Massif du Cinto, Capo al Berdato, versans SSW, pozzine de pente, 2320 m, 1.8.1969, Gamisans 2912 (fl.); cirque de Trimbolacciu, couloir de Pampanosa, pelouse, 1880 m, 6.8.1970, Gamisans 2913 (fl.)”].
Ochlopoa supina (Shrad.) H. Scholz & Valdés, Willdenowia 36: 662. 2006.
**Ill.**Pignatti (1982: 469).

##### Type.

“In summis alpibus Salisburgensibus (Mielichhoffer)”. (Possible isotype conserved in LE according to PAF 2018).

##### Flowering.

June to August (September).

##### Ecology.

Perennial grasslands in wet places; edaphically indifferent; 1200−3481 m a.s.l.

##### Distribution.

C and SW Europe, Apennines, Fennoscandia and NE Russia, extending to the Rif (Morocco). Pyrenees, Cantabrian Mountains, Central System and Sierra Nevada. **And. Port.**: (BA)? (Mi)? (TM)? **Spa.**: Av Ge Gr Hu L Le M Na P S. For a representative list of studied materials, see Suppl. material 1.

##### Notes.

Plants from the Sierra Nevada tend to have glabrescent floral parts (lemmas and paleas) or an indumentum that is restricted to the basal zone of the central nerve; this contrasts with plants from some peripheral populations of this mountain massif (e.g. Lugros, Dehesa del Camarate, 2200 m, GDAC 41005) and other Iberian populations, none of which are usually glabrous. Although clearly corresponding to *P. supina*, the Sierra Nevada plants also resemble those of *P. rivulorum* Maire & Trab., Bull. Soc. Hist. Nat. Afrique N. 15: 395. 1924; P. annua var. rivulorum (Maire & Trab.) Lit. & Maire in Jahand. & Maire, Cat. Pl. Maroc 1: 66. 1931; P. alpina subsp. atlantica (Trab.) Romo, Treb. Inst. Bot. Barcelona 11: 40. 1987, **syn. nov.**, non P. alpina var. atlantica Trabut in Maire, Mém. Soc. Sci. Nat. Maroc 7: 147. 1924; *Ochlopoa rivulorum* (Maire & Trab.) H. Scholz & Valdés, Willdenowia 36: 662. 2006], a tetraploid species (*n* = 14) endemic to Alto, Medio and Anti Atlas (Morocco), in that the flowers, as indicated by Maire (1955), are usually glabrous and rarely hairy at the base of the medial and marginal veins. The observed pattern in Sierra Nevada is perhaps simply infrapopulational variation.

#### 15. Poa bulbosa L., Sp. Pl. 70. 1753 subsp. bulbosa

Taxon classificationPlantaePoalesPoaceae

Poa bulbosa subsp. eu-bulbosa Hayek, Prodr. Fl. Penins. Balcan. 3: 259. 1932, nom. inval.
**Ill.**Ruiz (1991: 31, lam. III), Devesa (1987: 265).

##### Type.

“Habitat in Gallia” (lectotype designated by Meikle 1985, pg. 1742; restricted by Soreng 2000, pg. 255: Hasselquist, Herb. Linn. No. 87.57!).

##### Flowering.

January to July (December).

##### Ecology.

Pastures and grasslands in wet and nitrified soils, less frequently in ephemeral pastures and dry places; edaphically indifferent; 0−3100 m a.s.l.

##### Distribution.

Europe, SW, C and N Asia until W China, Africa and Macaronesia (Madeira and Canary Islands); introduced in the Americas, Australia and Pacific Islands. Entire Iberian Peninsula and Balearic Islands. **And. Port.**: Ag AAl BAl BA BB BL DL E Mi R TM. **Spa.**: A Al Ab Av B Ba Bu C Ca Cc (Cs) CR Co Cu Ge Gr Gu H Hu J L Le Lo Lu M Ma Mu Na Or O P PM[Mll (Me)] Po S Sa Sg Se So SS T Te To V Va Vi Z Za. For a representative list of studied materials, see Suppl. material 1.

##### Notes.

According to Bolòs and Vigo (2001: 380), reports of Poa bulbosa subsp. concinna (Gaudin) Hayek, Repert. Spec. Nov. Regni Veg. Beih. 30(3): 260. 1932 [*P. concinna* Gaudin, Agrost. Helv. 1: 196. 1811, basion., type: “Hab. in arenosis Valesiae inferioris, praecipue Seduni”; *P. perconcinna* J.R. Edmonson, Bot. J. Linn. Soc. 76: 330. 1978, type: based on *P. concinna* Gaudin] in E Spain are mistaken, as this taxon is actually only distributed from SE France to the Balkan Peninsula. That taxon differs from *P. bulbosa* by their smaller sizes and narrower leaves, 0.8–2.2 mm ligules and never-proliferating spikelets with 6–10 flowers. In a few peninsular populations, plants with spikelets bearing 9–10 flowers have been detected, but they coincide with *P. bulbosa* in all other characters.

Caryopses are formed in this species, but sexual reproduction is infrequent; more common propagation routes include the formation of bulbs at the base of the plant or bulbils at the inflorescence level. This latter phenomenon, pseudovivipary, is extraordinarily frequent in *P. bulbosa* and involves the formation of bulbils for vegetative multiplication in the place of normal flowers (“proliferated spikelets”; Ofir and Kigel 2014). These bulbils may or may not coexist with normal flowers on the same spikelet or plant or in the same population. The balance between clonal and sexual reproduction is controlled mainly by day length and temperature; short days and low temperatures usually promote proliferating inflorescences, whereas long days and high temperatures induce normal and seminiferous ones. The proliferating spikelets usually carry (1–) 2–3 bulbils and have deformed floral parts: the glumes are usually narrower, the lemma is typically long (up to 20 mm), thin and either glabrous or only hairy on the central and marginal veins and the palea is missing or fully integrated into the bulbil, similar to the lodicules. Stamens are also missing or very reduced in size. The bulbils tolerate desiccation, are dormant during the summer and are dispersed by wind and ants. Both the basal bulbs, which are also dormant during the summer and the inflorescence bulbils sprout at the peak of the winter rainy season (cf. Ofir and Kigel 2014).

Two varieties are distinguished in the flora. Plants with bulbils in the inflorescence are recognised as ***Poa bulbosa*** var. ***vivipara*** Koeler, Descr. Gram. 189. 1802 [Type: “prope Moguntiam in arenosis”; Poa bulbosa subsp. vivipara (Koeler) Arcang., Comp. Fl. Ital. 785. 1882; Paneion bulbosum var. viviparum (Koeler) Lunell, Amer. Midl. Naturalist 4: 222. 1915; Poa bulbosa f. vivipara (Koeler) Maire, Fl. Afrique N. 3: 86. 1955], a variety distributed in Europe, Africa and SW, C and N Asia to W China and introduced in the Americas, Australia and the Pacific Islands; it appears in practically all of the provinces of *Flora iberica* [**And. Port.**: AAl BAl BB BL E TM. **Spa.**: A Al Ab Av B Ba Bu Ca Cc CR Co Cu Ge Gr Gu H Hu J L Le Lo Lu M Ma Mu Na Or P PM[Mll] S Sa Sg Se So SS Te To V Va Vi Z Za], from sea level to 3100 m, with a preference for shady places. The other variety, ***Poa bulbosa*** var. ***bulbosa*** [*Poa crispa* Thuill., Fl. Env. Paris, ed. 2: 45. 1799, type: “Habitat in locis arenosis. Floret Maio”; *P. pasqualii* Heldr. ex Parl., Fl. Ital. 1: 343. 1850, nom. inval., pro syn.; P. bulbosa subsp. perligulata H. Scholz, Bot. Chron. 3: 17. 1983, Typus: “Italia: Insula Elba, marina di Campo, 12.4.1980, W. Lang w.n. (B)”; *P. perligularis* H. Scholz, Willdenowia 16: 404. 1987, non *Poa perligulata* Pilger, Notizbl. Bot. Gart. Berlin-Dahlem 11: 779. 1933; P. bulbosa f. variegata Font Quer, nom. nud., in sched. (MA 11272), **syn. nov.**; P. bulbosa f. minor H. Villar, nom. nud., in sched. (MA 156764, MA 156765), **syn. nov.**], is distributed throughout the range of the species and comprises plants in which the spikelets produce normal flowers; this variety is found mostly in provinces covered by *Flora iberica* [**Port.**: Ag AAl BAl BA BB BL DL E Mi R TM. **Spa.**: A Al Ab Av (B) Ba (Bi) Bu C Ca Cc (Cs) CR Co Cu Ge Gr Gu H Hu J L Le Lo Lu M Ma (Na) Or O P PM[(Ib) Mll (Me)] Po S Sa Sg Se So (SS) T Te To V Va Vi Z Za], where it ranges from sea level to 2895 m and generally thrives in exposed places.

Bulbs appear to have arisen in *Poa* at least twice and possibly as many as four times (Cabi et al. 2016). Taxa having bulbs are distributed in four clades: (1) P. supersect. Poa (*P. densa* Troitsky and *P. diversifolia* (Boiss. & Balansa) Hack. ex Boiss; (2) in or near P. supersect. Homalopoa (*P. pseudobulbosa* Bor); (3) N-clade (*P. pelasgis* H. Scholz); and (4) P. subgen. Ochlopoa sect. Arenariae (type: *P. bulbosa*) mixed with species of P. sect. Alpinae (type: *P. alpina*).

In Sierra de Mariola (Alicante), a population has been detected (MA 752991) in which the ligule of the upper leaves in some individuals is very short (1–1.5 mm), subtruncate and irregularly dentate or not.

#### 16. Poa ligulata

Taxon classificationPlantaePoalesPoaceae

Boiss., Voy. Bot. Espagne 2: 659. 1842.

**Figure 4.**
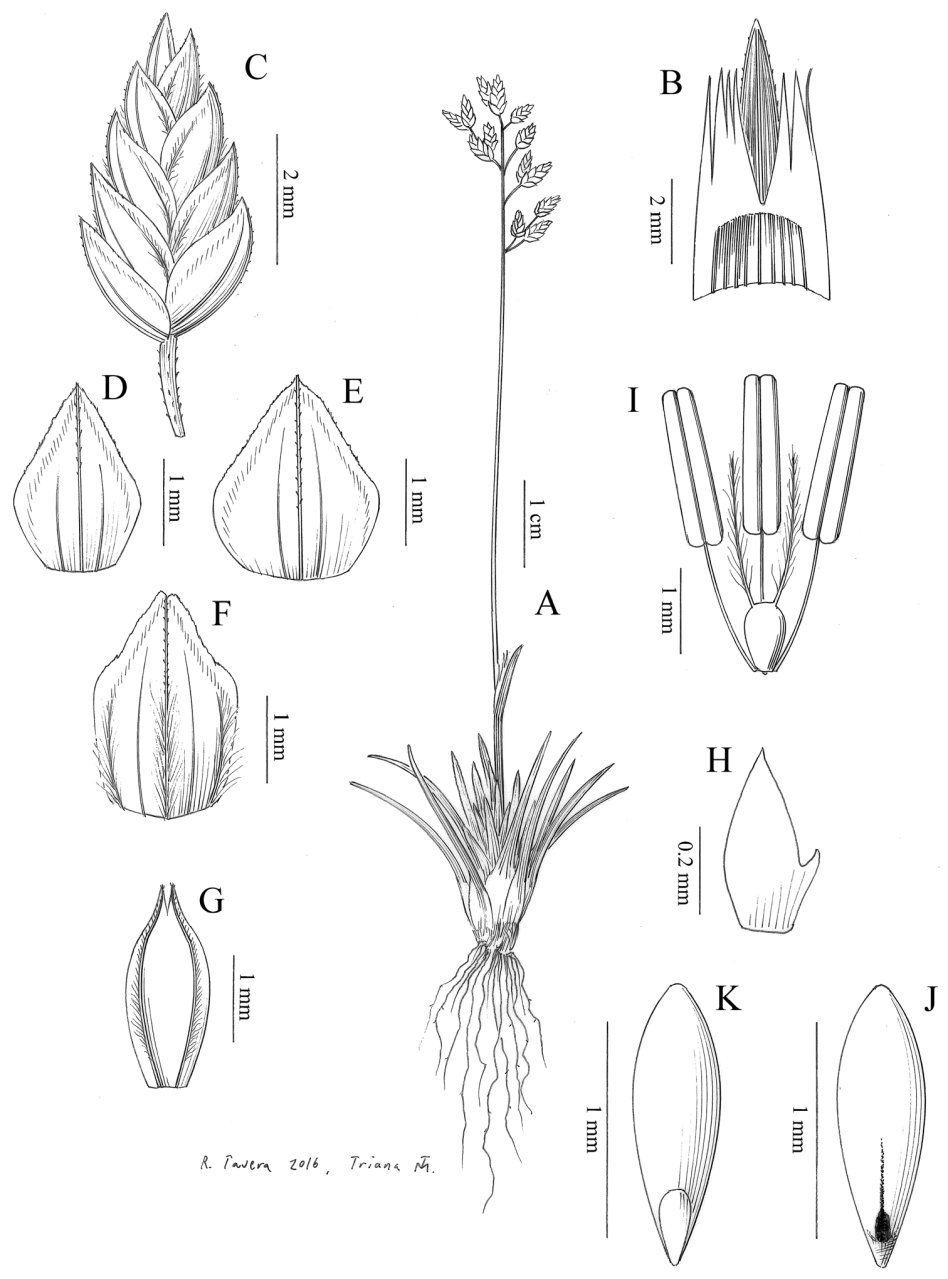
*Poa ligulata* Boiss. **A** Habit **B** Detail of the apex of the sheath and ligule, in adaxial view (upper leaf) **C** Spikelet **D** Lower glume, in abaxial view **E** Upper glume, in abaxial view **F** Lemma, in abaxial view **G** Palea, in abaxial view **H** Lodicule **I** Sexual verticils **J** Caryopsis, in adaxial view **K** Caryopsys, in abaxial view. Drawn from MA 414266 and MA 423181.

Poa concinna var. membranacea Boiss., Elench. Pl. Nov. 89. 1838, nom. subst. (Burdet et al. 1981 stated that they had not seen material corresponding to this taxon in herbarium G; however, they forget that this name has been replaced by P. ligulata and, therefore, the type material is the same).
Poa djurdjurae Trab. in Batt. & Trab., Fl. Alger 207. 1884. [Type: “Col de Tirourda (juin 1883)”].
Poa paui Font Quer, Iter Marocc. n. 34. 1928, nom. in sched. [Type: “Hab. in glareosis calc. montis Tisuka (Gomara), 2100 m. / alt.; 13 junii.” (lectotype designated here: “Dr. Font Quer. − Iter Maroccanum, 1928 / 34. Poa paui F. Q., sp. nov. / Hab. in glareosis calc. montis Tisuka (Gomara), 2100 m. / alt.; 13 junii. / Descr.: Poa ligulata affinis, sed folia subquadruplo lon-/ giora, ligulis subduplo elongatis et angustioribus; panicula laxa, / glumis virescentibus, spiculis majoribus.” (label printed): BC 70616, lower central specimen; isolectotypes designated here: BC 70616a, b, and c; MA 11378; GDA 31039)].
Poa ligulata var. paui (Font Quer) Maire, Bull. Soc. Sci. Nat. Maroc 11: 113. 1931.
Poa ligulata var. djurdjurae (Trab.) Maire, Bull. Soc. Hist. Nat. Afrique N. 22: 323. 1931.
Poa membranacea (Boiss.) C. Vicioso, Anales Jard. Bot. Madrid 2: 192. 1942, nom. illeg.; Anales Jard. Bot. Madrid, 6(2): 13. 1946.
Poa ligulata var. eu-ligulata Maire & Weiller in Maire, Fl. Afrique N. 3: 87. 1955, nom. inval.
Poa ligulata var. mauretanica Maire, Fl. Afrique N. 3: 88. 1955. [Type: “M. Beni Snassen au Ras Foughal! (E.); Moyen Atlas ! (M., J., E.) ; Rif (F.-Q., M.)”].
**Ill.**Bolòs and Vigo (2001: 378), Devesa (1987: 265), Fig. 4.

##### Type.

[Loc. ind. “Hab. in glareosis frigidis in sumâ Sierra Tejeda et in Sierra Nevada loco Corral dicto”] (lectotype designated by Hernández Cardona 1978, pg. 267, 358: top specimen on the right, G 00418689!, Herb.-Boiss.; isolectotypes: G 00418689a, b, c!, Herb.-Boiss.; ex herbier Reuter-Barbey, G 00418691!, 00418691a!; ex herbier De Candolle, G00418690!; vide Burdet et al. 1981, pg. 578).

##### Flowering.

(April) May to July.

##### Ecology.

Rocky places, pastures in protosols and stony places, on limestone and dolomite, less frequently on schist or gypsum; (700) 740−3200 m a.s.l.

##### Distribution.

Iberian Peninsula and NW Africa. E half of Spain, rarer towards the W. **Spa.**: (A) (Ab) Al Bu Ca Cc Cs CR Cu Gr Gu J Le Lo M Ma Mu Na (Or) P S Sg Se So Te To V Z. For a representative list of studied materials, see Suppl. material 1.

##### Notes.

This species is unmistakable because of its large ligules, which are very exerted, decurrent on the leaf sheath-especially those of the base and shoots-and pearly white, which makes its white-green tufts very striking. Its presence is indicated for the first time in the region of Extremadura (Spain).

#### 17. Poa alpina L., Sp. Pl. 67. 1753 subsp. alpina

Taxon classificationPlantaePoalesPoaceae

**Ill.**Soreng and Peterson (2012: 11, fig. I, A–E), Pignatti (1981: 473).

##### Type.

“Habitat in alpibus Lapponicis, Helveticis” (lectotype designated by Soreng 2000, pg. 254: Herb. Linn. No. 87.2!).

##### Flowering.

June to August (September).

##### Ecology.

Pastures in pine forests, fir woods, beeches and bushes, ruderalised hills and rocky plains, on limestone, schist, granite and slate; 1200−3150 m a.s.l.

##### Distribution.

Circumboreal: Europe, Asia, N America, locally at low elevations in NW Africa (Morocco). N Spain, Cantabrian Mountains, Pyrenees and the Iberian System. **And. Esp.**: (Av) B Bi Ge Hu L Le Lo Na O P S (Sa) So Te Vi. For a representative list of studied materials, see Suppl. material 1.

##### Notes.

A characteristic of this species is the size and shape difference of the ligule on the basal leaves and shoots—especially the oldest ones—compared with that of the upper leaves. In the first case, the ligule is tiny, with a more or less complete margin; in the second case, the ligule is noticeably larger, often with an irregular margin and sometimes even split into two or more parts.

**Figure 5.**
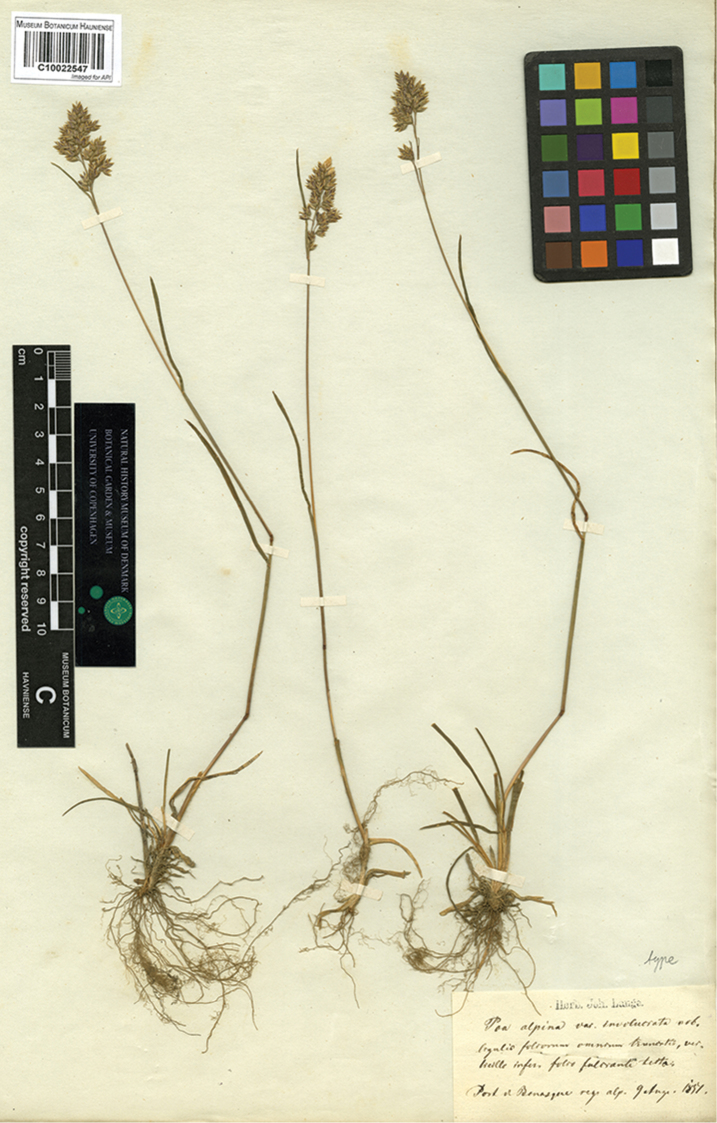
Lectotype of Poa alpina var. involucrata Lange (C 10022547). Reproduced with permission of the Natural History Museum of Denmark.

This is a very polymorphic taxon, with four recognised, sometimes intergrading varieties in the territory encompassed by our revision. Plant size, leaf stiffness and panicle contraction can vary extensively depending on altitude, exposure and soil type. The first of these varieties is ***Poa alpina*** var. ***alpina*** [*Poa frigida* Gaudin, Alpina 3: 33. 1808, type: “au-dessous du glacier de Plan-nové; dans la vallée de Bagnes etc.”; P. alpina var. frigida (Gaudin) Salisb., Flora 16(2): 473. 1833; P. alpina var. genuina Godr. in Gren. & Godr., Fl. France 3: 543. 1855, nom. inval.; P. alpina var. involucrata Lange, Pugill. Pl. Hispan. 47. 1860, **syn. nov.**, type: “In regione alpina Pyren. Hisp. ad Port de Benasque (9 Aug. c. fl.)!”, lectotype designated here: “Herb. Joh. Lange (printed) / Poa alpina var. involucrata nob. / ligulio foliorum omnium truncatis, ver. / -illegible- infer. folio -illegible- tetta / Port de Benasque reg. alp. 9 Aug. 1851. (manuscr.)”: C 10022547, specimen on the left), Fig. 5; *P. nuriensis alpina* Sennen, Bull. Soc. Bot. France 73: 677. 1926, nom. nud., **syn. nov.**; P. alpina subsp. digitata Beauverd, Bull. Soc. Bot. Genève, sér. 2, 26: 122, fig. 2a–g. 1936, type: “Hab. in locis apricis calidisque valleculae dictae “du Grand Tabuc” ad locum dictum “les Grangettes”, ca. 1800 m alt., supra thermis “Le Monétier de Briançon”, 15 Julii 1933, leg. J. Vergnet et G. Beauverd”; P. badensis subsp. multiflora sensu Rivas. Mart., Itinera Geobot. 15: 705. 2002, non P. alpina var. multiflora Gaudin, Fl. Helv. 1: 245. 1828; *P. nuriensis* Sennen, Pl. Espagne n. 4063. 1916, nom. nud., in sched. (BC 70515; MA 11298), **syn. nov.**]. This circumboreal taxon includes large plants having large, generally delicate, non-rigid leaves with non-thickened margins—or thickened less than 0.05 mm—and developed inflorescences that are only slightly condensed. This variety is typically found in sheltered, less-exposed locations and is widespread throughout the range of the species [**And. Spa.**: (Av) B Bi Ge Hu L Le Lo Na O P S (Sa) So Te Vi].

Under more adverse conditions, the plants are usually small and possess short, stiff leaves with non-thickened, or up to 0.05 mm thick, margins and very contracted panicles. These plants have been designated as ***Poa alpina*** var. ***brevifolia*** (Gaudin) Godr. in Gren. & Godr., Fl. France 3: 543. 1855 [Poa alpina subsp. brevifolia Gaudin, Fl. Helv. 1: 245. 1828, basion., type: “in M. Sempronio ad pylas vallis Ganter”], a variety distributed in mountains of C Europe and extended through the central and eastern Pyrenees [**And. Spa.**: B Hu L]. This pattern of variation is probably clinal and needs to be checked experimentally. The third recognised variety, ***Poa alpina*** var. ***molinerii*** (Balb.) Endl., Cat. Horti Vindob. 46. 1842 [*Poa molinerii* Balb., Elenco 85. 1801, basion., type: “Locis saxosis, et siccis prope Tenda reperta est, ac in hortum Taurinensem adlata ab eximio Ignatio MOLINERI, cujus triviale nomen imposui, utpote ejus stirpis inventore”], comprises plants having leaves with whitish, cartilaginous, thickened (0.1–0.15 mm) margins that, together with the middle underside vein, form a clear, visible contrast to the green leaf blade, the latter mostly flat or conduplicate and rigid. This taxon, is distributed in mountains of S and C Europe and, to date, only two populations of P. alpina var. molinerii have been detected for *Flora iberica*, one in **Andorra** (Coll de Ordino, on the way to Casamanya, 2100 m; MA 514862) and the other in **Spain** (Lérida, Clot del Munyidor, 2215 m; BC 877255).

Finally, ***Poa alpina*** var. ***vivipara*** L., Sp. Pl. 67. 1753. [Type: “An haec α. β. sequentis tantum varietas”; Lectotype designated by Soreng 2000, pg. 254: three left-hand culms, LINN-87.4!; *Poa vivipara* (L.) Willd., Enum. Pl. 103. 1809; P. alpina subsp. vivipara (L.) Arcang., Comp. Fl. Ital. 785. 1882; P. alpina f. vivipara (L.) B. Boivin, Naturaliste Canad. 94: 628. 1967], includes plants with pseudoviviparous spikelets. This variety, distributed mainly in N and C Europe, Greenland, Iceland and N America, has only been detected in a population in the territory covered by *Flora iberica* (**Spain**: Na), namely, it was collected in the valley of Roncal at 1600 m a.s.l (SEV 97164). The inflorescences of these plants conserved on this sheet show a great contrast of colours: glumes and lemmas of light green and straight or curved, dark green proliferations.

Another taxon described for the flora of Morocco is ***Poa alpina*** subsp. ***stenobotrya*** Maire (Bull. Soc. Hist. Nat. Afrique N. 33: 95. 1942), which is distinguished by its linear-lanceolate panicle and the presence of hairs at the base of the lemmas.

## Conclusions

In the territory covered by *Flora iberica*, the genus *Poa* is represented by 24 taxa (17 species, 1 subspecies and 8 varieties), mostly perennial. The majority of these taxa have broad global and/or European distributions, whereas two (*P. legionensis* and P. minor subsp. nevadensis) are Spanish endemics and two have restricted distributions (*P. ligulata*, Iberian–North African; *P. flaccidula*, Iberian–North African and the Balearic Islands, extending to Provence, France). The most widely distributed species are *P. bulbosa* and *P. annua*, reflecting their worldwide range. The provinces with the greatest representation of *Poa* are Huesca, Santander, Lérida and Andorra, all located in the N Iberian Peninsula, which are traversed by mountain systems and subjected to a temperate climate.

## Supplementary Material

XML Treatment for Poa pratensis L., Sp. Pl. 67. 1753 subsp. pratensis

XML Treatment for Poa legionensis

XML Treatment for Poa cenisia

XML Treatment for Poa chaixii

XML Treatment for Poa glauca Vahl in Oeder, Fl. Dan. 6(17): 3. 1790 subsp. glauca

XML Treatment for Poa nemoralis L., Sp. Pl. 69. 1753 subsp. nemoralis

XML Treatment for Poa compressa

XML Treatment for Poa laxa Haenke in J. Jirasek, Beobacht. Reis. Riesengeb. 118. 1791 subsp. laxa

XML Treatment for Poa minor

XML Treatment for Poa trivialis L., Sp. Pl. 67. 1753 subsp. trivialis

XML Treatment for Poa flaccidula

XML Treatment for Poa annua L., Sp. Pl. 68. 1753 subsp. annua

XML Treatment for Poa infirma

XML Treatment for Poa supina

XML Treatment for Poa bulbosa L., Sp. Pl. 70. 1753 subsp. bulbosa

XML Treatment for Poa ligulata

XML Treatment for Poa alpina L., Sp. Pl. 67. 1753 subsp. alpina

## Supplementary materials

Supplementary material 1 List of representative sheets for each province and taxon, excluding type material Data type: species data Explanation note: The total number of studied sheets is also indicated for each taxon. This is an open access article distributed under the terms of the Creative Commons Attribution License (CC BY 4.0), which permits unrestricted use, distribution, and reproduction in any medium, provided the original author and source are credited.Ana Ortega-Olivencia, Juan A. Devesa
