# Supplementary material for: Updated checklist of Poa in the Iberian Peninsula and Balearic Islands
Source: PhytoKeys. 2018 Jul 10;(103):27–60. doi: 10.3897/phytokeys.103.26029 (PMC6050348; doi:10.3897/phytokeys.103.26029)
Supplement: Supplementary material 1 — List of representative sheets for each province and taxon, excluding type material [file phytokeys-103-027-s001.docx]

**Supplementary material**

Authors: Ana Ortega-Olivencia, Juan A. Devesa

**Selected Specimens Examined**

List of selected sheets for each province and taxon, excluding type material. It is also indicated the total number of sheets studied for each taxon.

**1. *Poa pratensis*** L.

**Total number of studied sheets: 289 (var. *pratensis*), 258 (var. *angustifolia*) and 11 (var. *minor*).**

var. ***pratensis*. PORTUGAL. Alto Alentejo**: Elvas, 1946, illegible (MA 285035). **Beira Litoral**: Bussacco, Jun 1883, F. Loureiro (COI 10854). **Douro Litoral**: Porto, w.d., G. Sampaio n. 1901 (MA 11592). **Estremadura**: Alentejo litoral, sables de Trafaria, Jun/Jul 1892, J. Daveau (COI 10748). **Minho**: Esposende, Apúlia, dunes of Apúlia, 6 Jun 2010, A. Quintanar et al. AQ3871, Iter Lusitanicum (VI-2010) (MA 824700). **Tras-os-Montes e Alto Douro**: Bragança, between Bragança and Rica Fé, 20 Jun 1968, P. Silva & A.N. Teles n. 8198 (MA 196172).

**SPAIN.** **Albacete**: Alcaraz, 18 Jun 1984, near to Jardín River, J.M. Herranz (MA 330639). **Alicante**: Parcent, S. del Coll de Rates, 1 May 1994, J.X. Soler (MA 548466). **Almería**: Puerto de la Ragua, near Arroyo del Palancón, 15 Jun 1997, J. Lorite (GDA 52358). **Ávila**: Puerto del Pico, 22 May 1983, M. Luceño & P. Vargas, Herbario de Gredos (MA 773341). **Barcelona**: Porquerises, 26 Apr 1970, A.M. Hernández (MGC 1545). **Burgos**: Arlanzón, 12 Jul 1977, E. Fuentes (MA 520212). **Cáceres**: La Garganta, 29 Jun 1988, J.A. Devesa & M.C. Viera 3490/88 (UNEX 8414). **Castellón**: Segorbe, May 1889, C. Pau (MA 11572). **Ciudad Real**: Fuente de las Azadillas, 28 May 1933, J. González Albo (MA 11588). **Córdoba**: Cabra, ascending to the hermitage of the Virgen de la Sierra, J. Arroyo et al. (UNEX 1374). **Cuenca**: Chillarón de Cuenca, 15 May 1977, M. Costa Tenorio (MA 505175). **Gerona**: Ribes de Freser, 8 Jun 1969, J. Fernández Casas 1053 (MA 414309). **Granada**: Alfaguarilla, Alfaguara, w.d., A. Romero & C. Morales (GDAC 7509). **Guadalajara**: Puebla de Beleña, Laguna Grande, 8 Jun 1984, P. Pascual (SALA 60449). **Huesca**: Benasque, la Renclusa, 26 Jul 1955, P. Montserrat 885/55 (MA 211851). **Jaén**: Aldeaquemada, path of the Cimbarra, 27 May 1953, E.F. Galiano (SEV 6580). **León**: Puerto del Pontón, 19 Jun 1969, P. Montserrat 2864/69 (MA 211848). **Lérida**: Valle de Bohí, 6 Jul 1988, M. Ladero et al. (SALAF 23044). **Logroño**: Turruncún, Peña Isasa, 17 Jun 1972, A. Segura Zubizarreta (MA 355697). **Lugo**: Skirt of the Picos de Ancares, w.d., B. Merino (MA 11551). **Madrid**: Braojos, 2 Jun 1918, C. Vicioso (MA 1582). **Málaga**: Sierra Tejeda, Salares River, 26 Jun 1982, J.M. Nieto (MGC 20382). **Murcia**: Moratalla, Rincón de los Huertos, 24 Apr 1997, C. Aedo et al. 1161 IA (MA 591072). **Navarra**: NNE Espinal-Burguete, 18 Jun 1964, P. Montserrat (MA 211847). **Orense**: Serra do Invernadeiro, Suacenza, 21 Jun 1973, S. Castroviejo (SALA 6899). **Oviedo**: Oviedo, district of Vallobín, 20 Apr 1983, C. Aedo (MA 619053). **Palencia**: Fuentes de Nava, lagoon of La Nava, 12 May 1994, S. Cirujano (MA 624085). **Salamanca**: Salto de Almendra, May 1971, A. Asensi & B. Díez (MGC 1467). **San Sebastián**: Oleabzu, May 1895, M. Gandoger, Fl. Hispanica exsiccata n. 154 (MA 11548). **Santander**: Cábreces, 20 May 1923, E. Leroy (MA 646437). **Segovia**: Sebúlcor, Cantalejo road, 12 Jun 1983, T. Romero (MA 566435). **Soria**: Moncayo, Barranco de Agramonte, Agresa, 2 Jun 1933, L. Ceballos (MA 11563). **Tarragona**: track from Fredes to Monte Caro, base of La Mola del Boix, 8 Jun 1999, C. Navarro et al. CN2413 (MA 626798). **Teruel**: Fuente de Valdelinares, Sierra de Gúdar, 29 Jun 1955, E. Paunero (MA 184051). **Toledo**: Sangrera River, between Espinoso del Rey and Torrecilla de la Jara, 1 May 1990, J.L. Castillo & R. Cordero (MA 515146). **Valencia**: Titaguas, w.d., S. Clemente (MA 151385). **Valladolid**: Cigales, 22 May 2007, J.A. Lázaro Bello (MA 793819). **Zamora**: Cañizal, 10 May 1981, X. Giraldez (SALA 30635). **Zaragoza**: Salvatierra de Esca, Sierra de Illón, Esca River, 24 May 1982, A. Barra et al. n. 3211GF (MA 447888).

var. ***angustifolia*** (L.) Sm. **ANDORRA**. Parish of Sant Julià de Lòria, Cova de l'Ossa, 30 May 2004, C, Aedo et al., 10057 (MA 714813).

**PORTUGAl**. **Beira Litoral**: estrada d'Eiras, May 1873, A. Moller (COI 10755). **Minho**: Bualheira, Seixas, Jun 1885, A.R. Cunha (COI 10738). **Tras-os-Montes e Alto Douro**: Monte de S. Bartolomeu, Bragança, 5 Aug 1967, J. Paiva et al. (COI 10591).

**SPAIN**. **Albacete**: estate San Cristóbal near Alpera, 19 May 1968, P. Montserrat 1189/68 (MA 211856). **Alicante**: Vall de Laguar, S. de Migdia, near Manzaneda, 1 Jun 1996, J.X. Soler & M. Signes 4943 JXS (MA 587422). **Almería**: Bayárcal, Arroyo of Palancón, 15 Jun 1997, J. Lorite (GDA 52357). **Ávila**: San Pedro del Arroyo, 11 May 1960, P. Montserrat 2067/60 (MA 211804). **Barcelona**: Barcelona, Gavà, 9 Jun 2007, SBP 5441, S. Pyke (BC 907834). **Burgos**: Villaescusa de Roa, El Monte, 13 Jun 1981, J.L. Fdez. Alonso (MA 339737). **Cáceres**: Hermitage of the Humilladero, 30 May 1988, J.A. Devesa & R. Tormo (UNEX 12672-1). **Castellón**: Burriana, El Clot de la Mare de Déu, Apr 1986, orchard Clot, R. Roselló (MA 737020). **Ciudad Real**: Ciudad Real, 4 May 1933, J. González Albo (MA 11535). **Córdoba**: Cabra, ascent to the hermitage of the Virgen de la Sierra, 13 Jun 1984, J. Arroyo et al. (MA 453570). **La Coruña**: Fisterra, beach of Rostro, 11 May 1996, J. Amigo (MA 581600). **Cuenca**: Serranía de Cuenca, Jun 1962, J. Borja (MA 291748). **Gerona**: Banyoles, 25 Jun 2005, S. Pyke (BC 908119). **Granada**: Sierra de Baza, 15 Jun 1996, C. Salazar & F.B. Navarro (GDAC 40984). **Guadalajara**: Chillarón del Rey, 21 May 1970, F. Bellot et al. (SALA 6253). **Huesca**: Biescas, NE de Santa Elena, 21 May 1969, P. Montserrat (MA 211784). **Jaén**: Valdepeñas de Jaén, Puerto Pandera, 9 Jun 1964, P. Montserrat 1150/64 (MA 211862). **León**: Sierra del Teleno, Jul 1946, F. Bernis (MA 11566). **Lérida**: Seo de Urgel, Castellcintat, Prat de C. Guillen de Baix, 31 May 1958, P. Montserrat 802/58 (MA 211801). **Logroño**: Hervias, shores of the Laguna de Hervias, 30 May 1985, J.A. Alejandre (MA 339931). **Lugo**: Lugo, 31 May 1979, E. Carrera (MA 807536). **Madrid**: Rivas de Jarama, bank of the Jarama River, 17 Apr 1966, P. Montserrat 464/66 (MA 211794). **Málaga**: Tolox, Sierra de las Nieves, Pico Torrecillas, 13 Jun 1990, B. Cabezudo & J.M. Nieto (MGC 27549). **Murcia**: path Viejo de Monteagudo, 11 May 1978, A.M. Hernández (MA 209668). **Navarra**: Xabier, 8 Jun 1985, I. Aizpuru et al. (MA 364836). **Orense**: Serra do Invernadeiro, Suacenza, 21 Jun 1973, S. Castroviejo (MA 196174). **Oviedo**: Oviedo, district of Valobín, 23 May 1986, C. Aedo (MA 619072). **Palencia**: Cervera de Pisuerga, 19 Jun 1987, M.E. García (MA 711390). **Palma de Mallorca**: Lluch, 8 May 1949, P. Palau Ferrer - Plantes de Baleares n. 293 (MA 156033). **Salamanca**: Pelabravo, 4 May 1989, J. Pastor et al. (SALA 56984). **Santander**: Tama, 26 May 1986, C. Aedo (MA 619073). **Segovia**: Cerezo de Arriba, along the road to Riaza, 30 May 1965, P. Montserrat 287/65 (MA 211797). **Soria**: Herrera de Soria, 2 Jul 1984, M. Luceño (MA 371604). **Teruel**: Sierra de Gúdar, 29 Jun 1960, J. Borja (MA 170232). **Toledo**: Velada, near Guayerbas, 6 Apr 1983, T. Ruiz Téllez (SALAF 10435). **Valencia**: Utiel, May 1976, G. Mateo & L. Medir (MA 462707). **Valladolid**: Encinas de Esgueva, 13 May 1982, J.L. Fdez. Alonso (SALA 42362). **Vitoria**: Vitoria, w.d., A.P. Gredilla (MA 12459). **Zamora**: Moraleja del Vino, 27 May 1991, J.M. Velasco (SALA 58770). **Zaragoza**: Moncayo, 2 Jul 1964, s.r. (MA 549480).

var. ***minor*** Wahlenb. **SPAIN**. **Barcelona**: Barcelona, streets of Sants-Montjuic, 2 Apr 2001, S. Pyke (BC 832331). **Huelva**: Almonte, Matalascañas, 4 May1981, B. Valdés (SEV 98146). **Navarra**: Monte Irati, pass of Tapla to E of Goñiburo, 21 Jun 1960, P. Montserrat 364/60 (MA 211766). **San Sebastián**: Oiartzun-Altzibar, 14 May 1983, P. Catalán (MA 364244). **Santander**: Laredo, 21 May 1985, C. Aedo (MA 619052).

**2. *Poa legionensis*** (Laínz) Fern.-Casas & Laínz in Laínz

**Total number of studied sheets: 38**. **SPAIN**. **Ávila**: Fuente de los Barrerones, Sierra de Gredos, 8 Jul 1980, C. Aedo (MA 619081). **Cáceres**: Losar de la Vera, summit of Covacha, 12 Jul 1990, C.J. Valle & A. Amor (SALAF 23784). **León**: Cornón (near Lumajo, Villablino, León), 9 Jul 1963, M. Laínz, Herbarium Hispanicum Boreo-Occidentale (MA 410239). **Lugo**: Sierra de Ancares, Piornedo, 29 Jun 1982, S. Castroviejo et al. n. 6946SC (MA 548355). **Orense**: Casayo, road to Peña Trevinca, 28 Jul 1983, E. Bayón et al. n. 8846SC (MA 314664). **Oviedo**: Laguna de Arbás, Leitariegos, 2 Jul 1986, C. Aedo (MA 619080). **Salamanca**: La Hoya, Sierra de Béjar, Peña Negra, 10 Jul 1984, F. Amich et al. (MA 317779; SALA 35510).

**3. *Poa cenisia*** All.

**Total number of studied sheets: 64**. **SPAIN. Gerona**: Vall de Ribes, Nuria, 10 Jun 1955, J. Vives (BC 641495). **Huesca**: Aragüés del Puerto, Bernera, shady of the Bisaurín, 13 Aug 1970, P. Montserrat 5081/70 (MA 291615). **León**: Valverde de la Sierra, summits of Espiguete, P. Montserrat & L. Villar (BC 857184). **Logroño**: Pico de Urbión, 17 Sep 1977, A. Segura Zubizarreta (MA 356811). **Palencia**: Curavacas, 13 Aug 1990, C. Aedo (MGC 61625). **Soria**: summit of Pico Urbión, 23 Aug 1972, P. Montserrat & L. Villar (JACA 710072). **Zaragoza**: Moncayo, 17 Jul 1974, A. Segura Zubizarreta (MA 355689).

**4. *Poa chaixii*** Vill.

**Total number of studied sheets: 50**. **ANDORRA.** Port d'Envalira, 6.VI.2007, S. Pyke (BC 908096).

**SPAIN. Gerona**: Vall de Ribes, towards Coll de la Canya, 20 Aug 1972, J. Vigo (BC 611149). **Huesca**: Benasque, La Renclusa, ca. Xalet, 26 Jul 1955, P. Montserrat 881/55 (MA 216990). **León**: Busdongo, Rodiezmo, 10 Jul 1977, S.I. Laínz (MGC 61623). **Lérida**: Vall de Caneján, Arán, 18 Jun 1912, J.A. Soulié, Com. Sennen (BC 69953). **Logroño**: Sierra de la Hoz, 18 Jul 1931, F. Cámara (MA 11505). **Lugo**: Caurel, Bosque de Rogueira, 19 Jul 1935, P. Font Quer & W. Rothmaler (BC 149295, 1-3). **Oviedo**: Sierra del Páramo, Somiedo, 21 Jul 1978, J. Fernández Prieto (MA 453240). **Palencia**: Cervera de Pisuerga, 23 Jul 1975, A. Segura Zubizarreta (MA 356771). **Santander**: Barrio, Vega de Liébana, 2 Aug 1985, C. Aedo (MA 681557).

**5. *Poa glauca*** Vahl

**Total number of studied sheets: 6**. **SPAIN.** **Gerona**: Ribes de Freser, Collado Finestrelles, 3 Aug 1974, L. Villar (JACA 530774). **Huesca**: Bielsa, Pico de Urdiceto, 7 Sep 1997, J.V. Ferrández (JACA 374397).

**6. *Poa* *nemoralis*** L.

**Total number of studied sheets: 390 (**var. ***nemoralis*) and 80 (**var. ***rigidula*)**.

var. ***nemoralis***. **ANDORRA.** Andorra La Vella, 4 Jul 1992, C. Navarro CN-765 et al. (MA 525426).

**PORTUGAL.** **Beira Alta**: Guarda, barris de Farmaliçao, 17 Jun 1959, A. Fernandes et al. (COI 10736). **Douro Litoral**: Villa Nova de Gaya, Esteiro de Avintes, Jun 1900, G. Sampaio, Flora Lusitanica Exsiccata n. 1619 (MA 11477). **Tras-os-Montes e Alto Douro**: Bragança, Serra de Nogueira, Frago do Viborâo, 12 Jun 2010, C. Aedo et al. CA17429, Iter Lusitanicum (VI-2010) (MA 823667).

**SPAIN.** **Almería**: Ravine of Peña Horadada, 6 Jun 1996, J. Lorite (GDA 52372). **Ávila**: Puerto del Pico, 26 Jul 1982, D.S. Mata & P. Cantó (MAF 114701). **Barcelona**: Tarrasa, Jun, J. Cadevall (MA 11441). **Burgos**: Quintanar de la Sierra, 7 Jul 1914, P. Font Quer (MA 11433). **Cáceres**: Pico Jálama, 28 Jun 1988, J.A. Devesa & M.C. Viera (UNEX 12913-1). **Cádiz**: w.l., w.d., A.L. Cabrera (MA 151398). **Castellón**: Barranco del Azor, Vistabella del Maestrazgo, 21 Jun 1986, C. Fabregat (MA 489725). **Cuenca**: Tragacete de Valdemeca, 20 Jul 1979, G. López n. 1996 GF (MA 549988). **Gerona**: Ripollés, Pirineu, Nuria, Jul 1919, M. Gallardo (BC 853450). **Granada**: Sierra Nevada, Cañada de la Laguna de Aguas Verdes, 21 Aug 1980, A. Romero & C. Morales (GDAC 42529). **Guadalajara**: Peñalén, Hoz del Tajo, 19 Jul 1981, F. Muñoz 568FM (MA 548346). **Huesca**: Villanúa, 16 Aug 1982, J.A. Devesa & L. Villar (SEV 108183). **Jaén**: Arroyo of the Eruela, Sierra Mágina, 22 Jun 1925, J. Cuatrecasas (MGC 1465). **León**: Catoute, 26 Jul 1973, E. Hernández (SALA 22704). **Lérida**: Bohí, Muntanyó de Llacs, 8 Aug 1958, P. Montserrat (JACA 299088). **Logroño**: Niera de Cameros, 12 Jun 1907, C. Pau (MA 11434). **Lugo**: near Los Ancares, w.d., B. Merino (MA 11440). **Madrid**: Robregordo, 19 Jun 1918, C. Vicioso (MA 11463). **Málaga**: Tolox, P.N. Sierra de las Nieves, Cerro del Oso, 2 Jul 2014, B. Cabezudo, A.V. Pérez Latorre & F. Soriguer (MGC 79646). **Navarra**: Isaba, Barazea, 21 Jul 1986, I. Aizpuru & P. Catalán (MA 364245). **Orense**: Serra do Invernadeiro, mallada of Castiñeiro, 9 Jul 1973, S. Castroviejo (SALA 6900; MA 196162). **Oviedo**: Puerto Ventana, 3 Aug 1971, E.F. Galiano et al. (SEV 12583). **Palencia**: Piedrasluengas, 19 Jul 1971, M. Mayor & J. Andrés (SEV 24999). **Salamanca**: La Alberca, 2 Jul 1948, E. Paunero (MGC 1466). **San Sebastián**: Alto de Arlabán, 23 Jun1979, J. Loidi (MAF 123808). **Santander**: Peña Prieta, Vega de Liébana, 6 Aug 1985, C. Aedo (MA 619075). **Segovia**: Puerto Quesera, 19 Oct 1978, P. Blanco & R. Morales (SALA 85787). **Soria**: Montenegro de Cameros, 16 Jun 1977, A. Segura Zubizarreta (MA 356753). **Tarragona**: from Fresdes to Mount Caro, Ravine of Rafalgari, 8 Jun 1999, C. Navarro et al. CN-2447 (MA 626740). **Teruel**: between Nocito and Guara, 7 Aug 1970, J. Fernández Casas (MA 414265). **Toledo**: Montes de Toledo, 6 Jul 1977, A. Velasco (MAF 99747). **Valladolid**: Valbuena de Duero, 26 Jun 2010, Banks of the Douro River, J.A. Lázaro Bello (MA 827029). **Vitoria**: Campezo, San Román de Campezo, hill of Casilla, 16 Jun 1999, P.M. Uribe-Echebarría (SALA 100853). **Zamora**: Portilla de Padornelo, 19 Jul1973, E.F. Galiano et al. (GDA 18138). **Zaragoza**: Moncayo, 26 Jul 1978, A. Segura Zubizarreta (MA 356856).

var. ***rigidula*** Mert. & Koch. **ANDORRA.** Ranson, 29 Aug 2002, C. Aedo et al. CA 8583 (MA 700215).

**PORTUGAL.** **Tras-os-Montes e Alto Douro**: Braganca, Monte de S. Bartolomeus, 27 Jun 1908, A. Fernandes et al. (MA 285036).

**SPAIN. Almería**: Bayárcal, Arroyo Anchuelo, 31 Jul 1997, J. Lorite & C. Salazar (GDA 52370). **Ávila**: Sierra de la Paramera, next to Menamuñoz, 26 Jul 1982, J. Baranda et al. n. 334GF (MA 447893). **Burgos**: Hontanares, 6 Jul 1976, E. Fuentes (MA 520239). **Cáceres**: road of the Garganta, 10 Jun 1945, A. Caballero (SEV 6579). **Granada**: Pico Veleta, 12 Jul 1986, C. Aedo (MA 619085). **Huesca**: Panticosa-Bachimaña, 14 Jul 1965, S. Rivas-Martínez (MAF 103247). **Lérida**: Bohí-Espot, Sant Maurici National Park, Estany under Monartero, 25 Jul 1964, P. Montserrat (MA 211837). **Madrid**: Navacerrada, Jul 1982, J.L. Fernández Alonso (MA 519343). **Navarra**: Petilla de Aragón, 24 Jul 1988, I. Aizpuru et al. 3855JP (MA 459609). **Salamanca**: Topas, Jun, M. Rivas Mateos (MAF 28605). **Santander**: Canal del Vidrio, Áliva, 27 Jul 1952, E. Guinea, Phytotheca Selecta (MA 449617). **Segovia**: Cerezo de Arriba, Pico del Lobo, 16 Jul 1983, T. Romero (MA 566478). **Teruel**: Ravine of the Avellanar in Linares, Sierra de Gúdar, Jul 1958, J. Borja (MA 170229). **Zaragoza**: ascent to Moncayo, 17 Jul 1981, S. Castroviejo & Fdez. Quirós n. 6030SC (MA 547935).

**7. *Poa compressa*** L.

**Total number of studied sheets: 139**. **ANDORRA.** Sant Julia de Loria, Pont de la Marginada, 6 Jul 1992, S. Castroviejo 11937 et al., Iter Andorrano-Aranense, Jul 1992 (MA 512059).

**PORTUGAL.** **Tras-os-Montes e Alto Douro**: around Bragança, 12 Jun 1942, A. Carneiro (COI 10742).

**SPAIN. Alicante**: El Partagat, Serra de Aitana, Confrides, A. Rigual, Flora Lucentina (MA 371074). **Barcelona**: Santa María de Oló, 19 Jun 1970, J. Fernández Casas (MA 414287). **Burgos**: Quintanapalla, 19 Jun 1914, P. Font Quer (MA 11706). **Cuenca**: Headwaters of the Cuervo River, 8 Jul 1968, S. Rivas Goday & J. Borja (MAF 76302). **Gerona**: Ribes de Freser, 8 Jun 1969, J. Fernández Casas 10145 (MA 414269). **Granada**: Sierra Nevada, Hotel Santa Cruz, May 1980, A.T. Romero & C. Morales (GDAC 24577). **Guadalajara**: hill Casa Vicente, 3 Jul 1974, F. Muñoz Garmendia (MA 841758). **Huelva**: Aracena, 23 Jun 1966, J.A. Devesa (SEV 102153). **Huesca**: Hecho, forest house Sta. Ana de Siresa, 24 Jun 1969, P. Montserrat 3237/69 (MA 211820). **Jaén**: Riogazas, Sierra de Cazorla, 10 Jun 1982, A.M. Hernández (MA 291624). **Lérida**: Pallars Sobirà, Port de la Bonaigua, 26 Aug 1958, O. de Bolòs & O.H. Volk (BC 149121). **Logroño**: Puerto Piqueras, 6 Jul 1982, F. Amich (SALA 26582). **Madrid**: Navacerrada, Jul 1982, J.L. Fdez. Alonso (MA 519795). **Navarra**: Nazar, Peña Gallet, 16 Jun 1987, P.M. Uribe-Echebarría (SALA 48210). **Palencia**: pass of the Hoyo, 26 Jul 1987, M.E. García (MA 711392). **Salamanca**: Ciudad Rodrigo, 17 Jun 1978, E. Rico (SALA 13891). **Santander**: Lombraña, Polaciones, 10 Aug 1984, C. Aedo (MA 681559). **Segovia**: Navares de Enmedio, 17 Jul 1983, T. Romero (MA 566442). **Soria**: Piqueras, 26 Aug 1968, J. Fernández Casas (MA 414285). **Tarragona**: from Fredes to Mount Caro, under Mola del Boix, 8 Jun 1999, C. Navarro et al. CN-2416 (MA 626795). **Teruel**: Alcalá de la Selva, La Vega, 7 Jul 1957, P. Montserrat (MA 211819). **Valladolid**: Fompedraza, 11 Jul 1983, T. Romero (MA 566443). **Vitoria**: Leorza, 13 Jul1983, C. Aedo (MA 619061). **Zamora**: Fuentesauco, 11 Jun 1983, X. Giraldez (SALA 30736).

**8. *Poa* *laxa*** Haenke subsp. ***laxa***

**Total number of studied sheets: 40**. **ANDORRA.** Parish of Ordino, Tristaina, Port de l'Arbella, 25 Aug, C. Aedo et al. CA5253 (MA 629294).

**SPAIN. Gerona**: Vall de Ribes, Balandrau, 15 Aug 1972, J. Vigo & A. Anglada (BC 667377). **Huesca**: Benasque, Renclusa towards Maladeta, Aug 1992, S. Pyke (BC 908140). **Lérida**: Espot-Boí, near Collada del Muntanyó, 13 Aug 1978, A. Carrillo & J.M. Ninot (JACA 646791).

**9. *Poa* *minor*** Gaudin **a.** subsp. ***minor***

**Total number of studied sheets: 42**. **SPAIN. Gerona**: Cerdagne: Col de Finestrelles, 7 Aug 1916, F. Sennen, Plantes d'Espagne n. 2886 (MA 11338). **Huesca**: Faja de Pelay, 29 Aug 1969, J. Fdez. Casas (MA 414330). **Navarra**: Isaba, Larra, Pico de los Tres Reyes, 3 Sep 1971, L. Vilar & C. Dendaletche (JACA 10221571). **Oviedo**: El Fontán, Ubiña, 8 Aug 1990, C. Aedo (MA 619058). **Santander**: Picos de Europa, Peña Vieja, 4 Sep 1944, M. Martín & C. Vicioso (MA 11330).

**b.** subsp. ***nevadensis*** Nannf. in Font Quer, Exsicc. Fl. Iber. Select. Cent. 3: n. 201 (1935).

**Total number of studied sheets: 21**. **SPAIN. Granada**: Sierra Nevada, Cerro de la Alcazaba, 28 Aug 1923, P. Font Quer (GDA 31029).

**10. *Poa trivialis*** L.

**Total number of studied sheets: 548**. **PORTUGAL. Algarve**: Monchique, Jun 1887, A. Moller (COI 10636). **Alto Alentejo**: Serra de Portel, Horta do Derramado, 10 Jun 1982, J.V. Malato-Beliz & J.A. Guerra (UNEX 05504-1). **Beira Baixa**: Serra da Estrela, Penhas da Saúde, Jul 2003, F. Sales 03/190 (COI 66749). **Estremadura**: Sesimbra, Albufeira Arelva, 2 Jun 1971, J.V. Malato-Beliz & J.A. Guerra (UNEX 01782-1). **Beira Alta**: Ribeira do Torgal, near Odemira, 21 Jun VI.1981, A.T. Romero (GDAC 12385). **Beira Litoral**: Coimbra, Meco, 12 May 1995, M. Sequeira MS997 (MA 820100). **Douro Litoral**: Amarante, margin of the Tâmega River, 24 May 1940, A. Rozeira & M. Castro (SALA 26487). **Minho**: Viana do Castelo, Melgaço, S of Castro Laboreiro, abandoned village of Barreiro, 8 Jun 2010, J.L. Fernández-Alonso et al. JFA 29308, Iter lusitanicum (VI-2010) (MA 822137). **Ribatejo**: near Constancia, road in front of the nautical center, 5 May 2018, J. López (UNEX 36511). **Tras-os-Montes e Alto Douro**: Peredo de Bemposta-Mogadouro, S of Aldeia de P. Bemposta, margin of the Douro River, S of Falcueira, 30 May 1996, N. Marcos & M. Sequeira n. 2052 (MA 806668).

**SPAIN. Albacete**: Calar de la Sima, Yeste, 2 Jun 1990, J. Herranz et al. (MA 486877). **Alicante**: Ravine of the Cinc, Alcoy, 4 Jun 1961, A. Rigual (MA 371069). **Almería**: Berja, Sierra de Gádor, 24 Jul 1984, M. Luceño (MA 445637). **Ávila**: Puerto de Mijares, 13 Jun 1974, G. López & E. Valdés-Bermejo n. 1413 GF (MA 549703). **Badajoz**: Badajoz, May 1979, R. del Arco (UNEX 03034-1). **Barcelona**: Martorell, 23 May 1994, margin of the Llobregat River, I. Soriano (SALA 99844). **Burgos**: margin of the Urbión River, 18 Jun 1976, E. Fuentes (MA 520250). **Cáceres**: Brozas, 28 Apr 1987, A. Ortega & T. Ruíz (UNEX 13640-1). **Cádiz**: Algodonales, Sierra de Líjar, 13 Apr 1988, A. Aparicio 441/80 (GDA 13664). **Castellón**: between the Mas de la Cambra and La Estrella, Vistabella del Maestrazgo, 20 Jun 1987, C. Fabregat (MA 489768). **Ciudad Real**: Calzada de Calatrava, Hoz del Fresneda, 17 May 1995, J. Barrios Pérez & R. García Río (SALA 87802). **Córdoba**: Arroyo Martín Gonzalo, Sierra Morena, 17 Jun 1993, M. Melendo (GDAC 41585). **Cuenca**: Hoz de Beteta, 16 Jun 1979, G. López 1353GF (MA 549995). **Gerona**: between Malgrat and Palafolls, Tordera River, 1 Jun 1981, J.A. Devesa et al. 1791/81 (SEV 98145). **Granada**: Aguas Blancas River, near Quéntar, 18 May 1980, C. Morales & A.T. Romero (GDAC 7478). **Guadalajara**: Aldeanueva de Atienza, 12 Aug 1965, S. Silvestre (SEV 10939). **Huelva**: Sierra de Aracena, Higuera de la Sierra, La Junta. 15 May 1979, J. Rivera 4423/R (SEV 50073). **Huesca**: Ordesa, Gradas Soaso, 29 Aug 1969, J. Fernández Casas (MA 414305). **Jaén**: Benatae, Las Fuentes, Guadalimar, 19 Jun 1984, C. Fernández (JAEN 84-1502 A 84-1540) (MA 841502). **La Coruña**: Caramecheiro, Riveira. 14 May 1978, w.c. (SEV 106539). **León**: Sena de Luna, Jul 1990, M. Romero (MA 807213). **Lérida**: Vilanova de l'Aguda, 11 Jun 1985, J. Pedrol n. 545b JP (MA 419699). **Logroño**: El Rasillo de Cameros, w.d., I. Zubia (MA 11417). **Lugo**: Monforte, Areas, 10 Jun 1989, M.I. Romero (MA 530268). **Madrid**: Moncloa, May 1960, J. Borja (SALA 1751). **Málaga**: Montejaque, Gaduanes River, 18 Jun 1991, M. Osorio (MGC 29076). **Navarra**: Espinal, 18 Jun 1964, P. Montserrat 1754/64 (MA 291781). **Oviedo**: ascent to the Garganta, Jun 1980, T.E. Díaz (MGC 13999). **Palencia**: Palencia, channel of Aceña, 24 Jun 1989, C. López & A. Romero Abelló (MA 631968). **Palma de Mallorca**: Rafal Fort, Mahón, 11 May 1900, A. Pons Guerau (MA 11652). **Pontevedra**: El Casal, 25 May 1959, A. Ceballos (MGC 68928). **Salamanca**: Guijuelo, 7 Jun 1987, E. Rico & J. Serradilla (SALA 47698). **Santander**: Puerto del Escudo, 17 May 1960, F. Bellot & B. Casaseca (SALA 1750). **Segovia**: Sebulcor, 12 Jun 1983, T. Romero (SALA 40628). **Sevilla**: Cazalla de la Sierra, 16 May 1982, V. Bañez & J.L. García (SEV 99552). **Soria**: La Riba de Escalote, 1 Jul 1972, A. Segura Zubizarreta (MA 355719). **Toledo**: Gigüela River before Quintanar de la Orden, 13 May 1976, S. Cirujano (MA 548637). **Valencia**: Cañoles, Vallada, 21 May 1982, M. Palasí (MA 331762). **Valladolid**: Encinas de Esgueva, 22 Jul 1982, J.L. Fernández Alonso (SALA 42429). **Vitoria**: Maestu, Corres, path to San Román by the Casilla, 16 Jun 1999, P.M. Uribe-Echebarría (SALA 100854). **Zamora**: Villalonso, 28 Apr 1990, R. García Río (SALA 51967).

***Poa flaccidula*** Boiss. & Reut.

**Total number of studied sheets: 125**. **ANDORRA.** Parish of Sant Julià de Loira, borda del Germà, 1 Jun 2007, C. Aedo et al. 13865 (MA 756194).

**SPAIN.** **Alicante**: Sierra de Castalla, May 1962, A. Rigual (MA 651417). **Barcelona**: Obaga de les Espases, Olesa de Montserrat, 4 Jun 1994, A. M. Hernández, Plantae Catalaunicae Lectae in Annis 1980-2006, Exsiccata a Dr. Hernández Cardona facta n. 78 (UNEX 36440). **Burgos**: Hontoria del Pinar, 24 Jun 1959, P. Montserrat 801/59 (MA 291672). **Cádiz**: Grazalema, Cerro de San Cristóbal, 21 Jun 1989, B. Díez-Garretas (MGC 48225). **Castellón**: Cova Santa, 27 Jun 1987, M.B. Crespo (SALA 90620). **Ciudad Real**: Brazatortas, La Garganta, valley of La Garganta, 8 Jun 1997, R. García Río (MA 597267). **Cuenca**: from Tragacete to Valdemeca, 20 Jul 1979, G. López n. 1997GF (MA 549987). **Granada**: Sierra de la Sagra, Jun 1906, E. Reverchon, Plantes d'Espagne n. 656 (MA11495). **Guadalajara**: Poveda, Hoz del Tajo, 21 Jun 1974, F. Muñoz Garmendia (MA 841757). **Huesca**: Reservoir of Belsué, 11 Jun 1970, P. Montserrat 2208/70 (MA 291675). **Jaén**: Sierra de Cazorla, Navas de San Pedro, 24 Jun 1980, J.A. Devesa et al. (SEV 100997). **Lérida**: from La Seu to Sort, near parish of Ortó, 6 Jul 1986, P. & G. Montserrat (MA 479067). **Málaga**: Sierra de las Nieves, Los Quejigales, 14 Jun 1989, B. Díez-Garretas & A. Asensi (MGC 27483). **Palma de Mallorca**: Mallorca, Lluc, Puig den Galileu, 4 Jun 1998, R. Morales et al. 1823 RM (MA 618191). **Teruel**: Gúdar, 3 Jul 1957, P. Montserrat (MA 291674). **Toledo**: Los Yébenes, Sierra del Rebollarejo, 9 May 1998, L. Delgado et al. ER-6500 (SALA 96312). **Valencia**: Sierra Tortajada, Rincón de Adamuz, 3 Jun 1977, J. Vigo (SALAF 10023). **Zaragoza**: Jaraba, 11 May 1980, A. Segura Zubizarreta (MA 356849).

**12. *Poa annua*** L. subsp. ***annua***

**Total number of studied sheets: 708**. **ANDORRA.** Near Ordino, 29 May 2004, C. Aedo et al. (MA 714462).

**PORTUGAL.** **Algarve**: Olhão, Quinta de Marim, 30 Mar 1987, A. Moura (MA 395556). **Alto Alentejo**: Évora, Herdade da Mitra, 27 Mar 1984, J.V. Malato-Beliz & J.A. Guerra (UNEX 1740). **Baixo Alentejo**: between Cercal and Odemira, Arroyo Torgal, 19 Apr 1968, E. Paunero n. 5650 (MA 187988). **Beira Alta**: Sierra de la Estrella, 15 Jun 1982, J. Guerra (MGC 43114). **Beira Baixa**: Parque Natural da Serra da Estrela, in front N. Sra. da Boa Estrela statue, 22 Jun 2006, F. Sales et al. (COI 33138). **Beira Litoral**: Coimbra, Estaçao Velha, 12 Apr 1956, A. Matos & A. Marques (MA 187462). **Douro Litoral**: Porto, 6 Apr 1971, w.c. (MA 787484). **Estremadura**: Sesimbra, Lagoa de Albufeira, 19 Apr 1988, A. Moura (MA 479500). **Minho**: Viana do Castelo, Melgaço, S of Castro Laboreiro near abandoned village of Barreiro, 8 Jun 2010, J.L. Fernández-Alonso et al. JFA29374, Iter Lusitanicum (VI-2010) (MA 822042). **Tras-os-Montes e Alto Douro**: Bragança, Serra da Nogueira, road to Nossa Senhora da Serra, 12 Jun 2010, C. Aedo et al. CA17579 (MA 823374).

**SPAIN. Alicante**: Vall de Gallinera, Castell de Gallinera, 29 Oct 1996, J.X. Soler & M. Signes 5243 JXS (MA 587523). **Almería**: Adra, Pago del Lugar, 11 Dec 1982, A. Ortega & A.B. Robles (GDAC 27444). **Ávila**: Ramacastañas, 1 Mar 1987, F. Gómez et al. (MA 783939). **Badajoz**: Almendral, 2 Apr 1987, J.A. Devesa & M.C. Viera 557/87 (UNEX 13591). **Barcelona**: Porquerises, 26 Apr 1970, A.M. Hernández (MGC 1544). **Bilbao**: Erguiriñao, Gorbea, 26 Jul 1948, E. Guinea, Phytotheca Selecta n. 2455 (MA 553420). **Burgos**: Frías, 21 Oct 1984, I. García Mijangos (MA 549823). **Cáceres**: Puente de la Bazagona, 22 Apr 1988, A. Ortega & J.A. Devesa 2122/88 (UNEX 13613). **Cádiz**: Chiclana, 21 Apr 1980, F. Amor & A. Barroso (SEV 97173). **Castellón**: Columbretes, Illa Grossa, near La Caserna, 27 Apr 2006, C. Aedo et al. 12650 (MA 771576). **Ciudad Real**: Casas del Río, banks of the Bullaque River, 20 Mar 1993, Díaz Fdez. et al. (MA 629843). **Córdoba**: Los Blázquez, Zújar region, 22 Apr 1982, J.A. Devesa et al. (SEV 97175). **Cuenca**: Buendía, 8 Apr 1976, M. Costa (MA 504457). **Gerona**: Roca de La Malé, Nuria shrine, 2 Sep 1944, P. Montserrat (MA 549476). **Granada**: Guadix River, Jerez del Marquesado, 14 Apr 1995, C. Salazar (GDAC 41006). **Guadalajara**: Molina de Aragón, 1 Apr 1998, J.M. Pisco García 98/6 (MA 638903). **Huelva**: El Rocío, La Algaida, 19 May 1984, J.A. Devesa & S. Talavera (UNEX 1379). **Jaén**: Sierra de Cazorla, Pico Cabañas, 24 Jun 1980, J.A. Devesa et al. (SEV 97180). **La Coruña**: Artes, 22 Apr 1978, w.c. (SEV 97207). **León**: Santa Eulalia, 5 Aug 1979, M.J. Díez (SEV 97212). **Lérida**: near Bellvis, 3 Aug 1987, J. Pedrol 2148JP (MA 483648). **Logroño**: Dehesa de Ausejo, 29 May 1988, J. Arizaleta et al. 3166 JP (MA 438370). **Lugo**: around the city, 27 Jun 1992, E. Carreira (SALA 56878). **Madrid**: Aranjuez, 2 Jun 1857, J. Isern (MA 11179). **Málaga**: Málaga, Campus de Teatinos, 2 May 2003, M. Melgar (MGC 67821). **Murcia**: Huerta de San Antonio Abad, Cartagena, 16 Dec 1900, F. Jiménez (MA 11189). **Navarra**: Huarte-Araquil, 4 May 2002, E. Robles (MGC 67365). **Oviedo**: Oviedo, Feb 1981, C. Aedo (MA 619066). **Palencia**: Golobar, near Brañosera, Jun 1988, M. Laínz (MA 465549). **Palma de Mallorca**: Llucmajor, Cala Pi, 7 Jun 1998, M. Velayos et al. MV9046 (MA 619112). **Pontevedra**: Lourizán, 21 Jan 1989, A. Estévez (SALA 115809). **Salamanca**: Montemayor del Río, 16 Mar 1985, E. Rico & A. Guillén (SALA 36025). **San Sebastián**: Hernani, Mar 1895, M. Gandoger, Fl. Hispanica Exsicc. n. 26 (MA 11163). **Santander**: Santander, Magdalena Peninsula, 27 Dec 2008, C. Aedo 16023 (MA 777771). **Segovia**: Sacramenia, 6 Oct 1985, T. Romero (MA 566444). **Sevilla**: El Real de la Jara, 29 Apr 1984, J. Arroyo et al. (UNEX 1450). **Soria**: Quintana Redonda, 21 May 1977, A. Segura Zubizarreta (MA 356809). **Tarragona**: Sierra del Montsiá, San Carles de la Rapita, Font de Burgá, 11 Jun 1999, M. Velayos et al. 9382 (MA 626520). **Teruel**: Monreal del Campo, May 1897, J. Benedicto (BC 853458). **Toledo**: Talavera de la Reina, 21 Jun 1969, E. Valdés Bermejo (MA 548604). **Valencia**: Játiva, hill of the castle, 7 Apr 1968, F. Bellot & M.E. Ron (GDAC 34546). **Valladolid**: Encinas de Esgueva, 5 Apr 1983, J.L. Fernández Alonso R-353 (MA 346916). **Vitoria**: Salinas de Añana, Sobrón, La Playa, banks of the Ebro River, 28 Apr 1982, P. Uribe & J.A. Alejandre n. 866-82 (MA 452029). **Zamora**: Calzada de Tera, 4 Aug 1990, R. García Ríos (SALA 51963). **Zaragoza**: Calatayud, May 1910, C. Vicioso (MA 11168).

**13. *Poa infirma*** Kunth in Humb., Bonpl. & Kuntz

**Total number of studied sheets: 184**. **PORTUGAL. Algarve**: Faro, Mar 1919, F. Mendonça (MA 11156). **Alto Alentejo**: Altei do Ghâo, Candelaria de Altei, 17 Mar 1955, J.V. Malato-Beliz & A.F. Raimundo (MA 285053). **Baixo Alentejo**: Sines, S de S. Torpes, near Vale de Figueira beach, 26 Mar 1981, L.A. Grandvaux (MA 414849). **Beira Alta**: Tabuaço, May 1887, C.J. de Lima (COI 10728). **Estremadura**: Serra de Arrabida, Mata do Solitario, 17 Apr 1968, J.V. Malato-Beliz et al. n. 5485 (MA 285037). **Minho**: Vila Nova de Cerveir, Gondarém, between Estalagem Boega and El Calvario, 5 Jun 2010, A. Quintanar et al. AQ3742 (MA 824858). **Tras-os-Montes e Alto Douro**: Mirandela, Lameirâo, 4 May 1961, J.V. Malato-Beliz et Ruivo (MA 285055).

**SPAIN. Alicante**: Gata de Gorgos, 1 Apr 1975, A.M. Hernández (MGC 2167). **Almería**: El Ejido de Dalías, Feb 1970, J. Fernández Casas (MA 420452). **Ávila**: Hoya de la Laguna de Gredos, 26 Jun 1915, H. Villar (MA 156760). **Badajoz**: Medellín, 21 Mar 2001, C. Aedo et al. CA 6006 (MA 691293). **Barcelona**: Font del Bocallá, Serra de Collserola, 29 Mar 1975, A.M. Hernández (MGC 2219). **Cáceres**: Puerto de Miravete, 3 Mar 1988, J.A. Devesa & R. Tormo (MA 521906). **Cádiz**: El Bosque, road to Ubrique, 4 Mar 1983, A. Aparicio et al. (MA 460708). **Ciudad Real**: Fuencaliente, valley of the Navalmanzano River, 17 Mar 1996, R. García Río (MA 597256). **Córdoba**: Los Pedroches, Dos Torres, margins of the Arroyo Herrumbrosa, 2 May 1976, J.A. Devesa (SEV 35087). **Gerona**: Llers, 12 Mar 1908, F. Sennen, Plantes d’Espagne n. 606 (MA 11143). **Granada**: Salobreña, 7 Feb 1980, A.T. Romero & C. Morales (GDAC 24586). **Huelva**: Campo Abajo, Cartaya, El Chaparral, 20 Apr 1982, J.M. Polo & C. Norman (SEV 97635). **Huesca**: Ballobar, 19 Mar 1975, A.M. Hernández (MGC 2369). **Jaén**: Andújar, La Fuentecilla, 31 Mar 1993, T. Carrera (MA 652263). **Logroño**: Navarrete, May, I. Zubia (MA 11144). **Madrid**: Colmenar Viejo, 11 Apr 1966, P. Montserrat (MA 211816). **Málaga**: Nerja, Miel River, Jul 1984, J. Jara Ruiz (MGC 35147). **Oviedo**: Bojo, Allande, 5 Apr 1987, C. Aedo (MA 619060). **Salamanca**: Ledesma, 24 Mar 1978, J. Sánchez (SALA 19003). **Palma de Mallorca**: Mallorca, Puig de ca’n Enric, Andratx, 2 Jan 2000, M. Mayol & L. Sáez LS-5302 (MA 633804). **Santander**: Tolibes, near Potes, 10 Mar 1988, C. Aedo (MA 619087). **Segovia**: Aldeonsancho, 26 May 1985, T. Romero (SALA 40613). **Sevilla**: Castilleja de la Cuesta, 12 Mar 1968, E.F. Galiano et al. (SEV 97643). **Teruel**: in Aragonia australis, w.d., F. Loscos (COI-Willk 36521). **Toledo**: Alcaudete de la Jara, 17 May 1967, P. Montserrat 422/67 (MA 291799). **Valencia**: Valencia, 20 Jul 1977, A. Salcedo (SEV 997640). **Valladolid**: Mucientes, 3 May 2008, J.A. Lázaro Bello (MA 827032). **Zamora**: El Campillo, El Castillo, 17 Mar 1990, R. García Rios (SALA 51964).

**14. *Poa supina*** Schrad.

**Total number of studied sheets: 47**. **ANDORRA.** Parish of Canillo, Ransol valley, Estany dels Meners de la Coma, 26 Jul 2005, C. Aedo et al. 12183 (MA 732200).

**SPAIN. Ávila**: Pico Zapataero, Fuente del Ama, 28 Jul 1982, D.S. Mata & P. Cantó (MAF 114699). **Gerona**: Catalogne: Pyrénées, Nuria to Monsanto, 4 Sep 1916?, F. Sennen, Plantes d'Espagne (BC 70479). **Granada**: Lugros, Dehesa del Camarate, 9 Aug 1996, C. Salazar (GDAC 41005). **Huesca**: Ibón Viejo, Bernera, Valle de los Sarrios, Ansó, 12 Aug 1988, P. Montserrat & L. Villar (MA 479069). **Lérida**: Arán valley, near Estanh Long de Liat, 8 Oct 2006, C. Aedo et al. 13509 (MA 745227). **Madrid**: Sierra de Guadarrama, ascending to Peñalara, Jul 1969, S. Rivas-Martínez (MAF 116760). **Navarra**: Valle del Roncal, 2 Aug 1987, M. Luceño et. al PV 2481 (MA 356576). **Palencia**: Valdecebollas peak, 9 Aug 1987, C. Aedo (MA 619082). **Santander**: Fuente del Chivo, Polaciones, 18 Jul 1989, C. Aedo (MA 619050).

**15. *Poa bulbosa*** L. subsp. ***bulbosa***

**Total number of studied sheets: 667 (var. *bulbosa*) and 506 (var. *vivipara*)**.

var. ***bulbosa***. **PORTUGAL.** **Algarve**: Serra de Monchique, 23 Apr 1968, F. Bellot & B. Casaseca (MA 187907). **Alto Alentejo**: Mourao, road of Villanueva del Fresno and Mourao, 21 Mar 2001, S. Castroviejo et al. (MA 691591). **Baixo Alentejo**: bridge over the Múrtiga River, N of Moura, 21 Mar 2001, C. Aedo et al. n. 111VV01 (MA 691400). **Beira Alta**:

Serra da Estrela, Manteigas, Penhas Douradas, 2 May 1994, E. Rico et al., VI Itinera Mediterranea n. 1487 (MA 718944). **Beira Baixa**: P. Natural da Serra da Estrela, Penhas da Saúde, 4 May 2005, F. Sales et al. (COI 33214). **Beira Litoral**: Vilarinho de Lousâo, Soito, 24 Mar 1961, A. Fernandes et al. (COI 10666). **Douro Litoral**: Aveiro, Arouca, estrada Arouca -Alvarenga, ca. 1 km de Alvarenga, 25 Mar 1980, A. Marques (COI 10656). **Estremadura**: Setubal, Apr 1900, A. Luisier (COI 10680). **Minho**: Povoa de Lanhoso, Apr 1895, G. Samapio (COI 10701). **Ribatejo**: Setil, Monte da Quinta das Malhadas, 9 Apr 1946, Garcia & Sousa (COI 10678). **Tras-os-Montes e Alto Douro**: Mirandela, 29 Mar 1942, A. Rozeira (MA 183886).

**SPAIN. Albacete**: Bienservida, 6 Apr 1993, I. Álvarez IA147 & N. Yagüe (MA 609661). **Alicante**: Villena, lower part of Cabezo Redondo, 7 Apr 1957, A. Rigual (MA 371033). **Almería**: Laujar, Andarax River, 17 Apr 1981, P. Sánchez (GDAC 10658). **Ávila**: valley of the Corneja River, 16 May1986, J. Estrada (MA 505921). **Badajoz**: Santa Amalia, Guadalo River, 20 Mar 2001, J.J. Aldasoro 1210 (MA 691643). **Burgos**: El Parral, near Burgos, May1914, P. Font Quer (MA 11272). **Cáceres**: Navalvillar de Ibor, between Navalvillar de Ibor and Guadalupe, J.P. Carrasco & R. Tormo 1794/88 (MA 521937). **Cádiz**: Benamahoma, 27 Mar 1983, A. Aparicio et al. (MA 467248).

**Ciudad Real**: Solana del Pino, Alharín, 19 Mar 1998, w.c. (MA 712643). **Córdoba**: district of Los Pedroches, Pozoblanco, Cortijo de La Romana, 2 Apr 1976, J.A. Devesa (SEV 35096). **La Coruña**: Riveira, 29 Apr 1979, w.c. (SEV 97603). **Cuenca**: from Tragacete to Valdemeca, 25 May 1974, A. González & G. López (MA 415639). **Gerona**: Santa Coloma de Farners, 7 May 1970, A.M. Hernández (MGC 1535). **Granada**: Sierra de Baza, Prados del Rey, 8 Jul1984, J. Torres et al. (GDAC 26253). **Guadalajara**: Valverde de los Arroyos, w.d., E. Monasterio-Huellín 575M-H (MA 531160). **Huelva**: Sierra de Aracena, between Valdeflores and Higuera de la Sierra, 24 Feb1978, J. Rivera (SEV 50066). **Huesca**: Sierra de Guara, Pontón, 9 Jul1902, C. Pau (MA 446806). **Jaén**: Santiago de la Espada, Calar de las Palomas, 3 Jun1983, C. Soriano (MA 462243). **León**: Santa Colomba de Somoza, Apr 1946, F. Bernis (MA 11265). **Lérida**: Cubells, 30 Apr1983, J. Pedrol (MA 419332). **Logroño**: Logroño, 3 May, w.c., Herbario Español (MA 11273). **Lugo**: between Monforte de Lemos and Quiroga, 11 Apr 1977, S. Castroviejo 501 (MA 447589). **Madrid**: Aranjuez, Mar de Ontígola, 24 Apr 1979, M.J. Díez (SEV 97157). **Málaga**: Sierra de Aguas, Carratraca, Pico Aguas, 17 May1972, G. López (MA 553320). **Orense**: Sierra do Invernadeiro, Vega de Meda, 20 Apr 1973, S. Castroviejo (MA 196148). **Oviedo**: Cueva Santa, n. 33, 3 Sep 1886, w.c. (MA 446781). **Palencia**: Villamedianba, Monte del Rey, 2 May1987, C. López & A. Romero Abelló (MA 627175). **Palma de Mallorca**: Palma, 10 Mar 1947, P. Ferrer (MA 347238). **Pontevedra**: Lourizán, 4 May 1951, A. Rodríguez (MA 200297). **Salamanca**: Barbadillo, 25 Apr 1989, M. Luceño & P. Vargas (MA 464176). **Santander**: Pozas de Llorosa, Áliva, 19 Jul 1950, E. Guinea, Phytotheca Selecta (MA 449628). **Segovia**: Aguilafuente, Puente Cega, 13 May 1998, R. Alegría (MA 755045). **Sevilla**: between Carmona and La Luisiana, 17 Apr 1975, B. Cabezudo & S. Talavera 2707/75 (MA 356848). **Soria**: Miño de Medinacelli, 11 May 2004, C. Aedo et al. AQ 1057 (MA 733271). **Tarragona**: Batea, 3 May 1925, C. Pau (MA 11277). **Teruel**: Monreal del Campo, w.d., J. Benedicto (MA 446782). **Toledo**: Sangrera River, between Espinoso del Rey and Torrecilla de la Jara, 1 May 1990, J.L. Castillo & R. Cordero (MA 515138). **Valencia**: Venta del Moro, 11 May 1984, G. Mateo (MA 388920). **Valladolid**: Encinas de Esgueva, May 1982, J.L. Fernández Alonso R-361 (MA 339730). **Vitoria**: Laguardia, 1 May 1983, J.A. Alejandre (MA 268965) **Vitoria**: Laguardia, 1 May 1983, J.A. Alejandre (MA 268965). **Zamora**: Escober, El Badendin, 10 Apr 1996, P. Bariego (MA 651132). **Zaragoza**: Calatayud, 8 Apr 1905, C. Vicioso (MA 11269).

var. ***vivipara*** Koeler. **ANDORRA.** Near Ordino, 29 May 2004, C. Aedo et al. n. 9952 (MA 714457).

**PORTUGAL. Alto Alentejo**: Serra de S. Mamede, Salâo Frio, 28 Apr 1994, VI Itinera Mediterranea n. 1027 (MA 716809). **Beira Alta**: from Guarda to Vilar Formoso, 19 Apr 1952, A. Fernandes et al., Fl. Lusitanica Exsiccata n. 4607 (MA 285040). **Beira Baixa**: Serra da Estrela, Castelo Branco, besides Nave de Santo António, 15 Apr 1987, W.O. van der Knaap & J.F.N. van Leeuwen (COI 10725). **Beira Litoral**: Serra da Louzâ, Apr 1911, M. Ferreira, Fl. Lusitanica Exsiccata (MA 11238). **Estremadura**: Vale do Forno, 22 Apr 1943, J.G. Garcia (COI 10711). **Tras-os Montes e Alto Douro**: Meirandela, Romeu, Quadraçal, 23 Apr 1955, J.V. Malato-Beliz et al. n. 1997 (MA 285038).

**SPAIN. Albacete**: Peñas de San Pedro, Castillo de Peñas, 29 Apr 1977, J. Fernández Casas 1528 et al. (MA 395761). **Alicante**: Cocentaina, Serra de Mariola, Alt del Montcabrer, 10 May 2004, L. Serra 6861, J. Pérez & J.V. Andrés (MA 752991). **Almería**: Sierra Nevada, Puerto de la Ragua, summit of pass W of Chullo, 12 Apr 1967, P.W. Ball et al. 12.VI.1967 (MA 187076). **Ávila**: valley of the Corneja River, 17 Apr 1985, M. Carrasco et al. (MA 506479). **Badajoz**: Peraleda del Zancejo, between Peraleda del Zancejo and Zalamea, Sierra de los Argallanes, 10 Mar 1988, J.P. Carrasco & A. Muñoz 588/88 (MGC 40098). **Barcelona**: Barcelona, w.d., Herbarium Colmeiro (MA 11226). **Burgos**: Covarrubias, Los Valles, 22 May 1999, M. Rodrigo Juarros (MA 750092). **Cáceres**: Valverde del Fresno, 12 May 1988, J.P. Carrasco & A. Ortega 2634/88 (MA 522902). **Cádiz**: Grazalema, Puerto de las Palomas, May 1991, V.E. Martín Osorio (MGC 33665). **Ciudad Real**: San Lorenzo de Calatrava, Cerro de la Encantada, 26 Mar 2002, A. Quintanar (MA 729511). **Córdoba**: Fuente Alhama, 16 Apr 1982, J. Arroyo (SEV 84545). **Cuenca**: Serranía de Cuenca, Los Torcales, 14 May 1972, S. Rivas Goday et al. (MA 291606). **Gerona**: Palamós, San Cebrián dels Alls Can Dalman, 11 Apr 1949, P. Montserrat (MA 291591). **Granada**: Sierra de Baza, Venta del Vicario, 14 May 1984, J. Torres et al. (GDAC 26252). **Guadalajara**: Cantalojas, 17 May 1992, F. Martínez García (MA 657693). **Huelva**: Sierra de Aracena, between El Patrás and Zalamea la Real, 11 Mar 1980, J. Rivera (SEV 53518). **Huesca**: Bielsa, Circo de Pineta, 24 Apr 1955, E. Paunero (MA 184030). **Jaén**: Pontones, Aroca, 10 May 1980, S. Pajarón (MA 512703). **León**: Torrebarrio, San Emiliano, 10 May 1984, C. Aedo (MA 619064). **Lérida**: Cubells, 7 May 1983, J. Pedrol (MA 419374). **Logroño**: Ojacastro, Artaso Ravine, 14 May 1999, G. García-Baquero 1561 (SALA 100762). **Lugo**: Lugo, 4 Jun 1986, E. Barreira (MA 807535). **Madrid**: El Escorial, 17 Jun 1965, B. Valdés (SEV 54131). **Málaga**: Antequera, Sierra del Torcal, 24 Apr 1966, E.F. Galiano & Novo (MA 183267). **Murcia**: Moratalla, Rincón de los Huertos, 24 Apr 1997, C. Aedo et al. 1160 IA (MA 591074). **Navarra**: Metauten-Ganuza, Sierra de Lokiz, 17 Apr 1984, P. Catalán & I. Aizpuru n. 320.84 (MA 364905). **Orense**: Rubiá, under Pardollán, Peñarrubia reservoir, 20 Apr 1991, J. Amigo & M.I. Romero (MA 503414). **Palencia**: Peña Cueto, 1 May 1987, M.E. García (MA 711391). **Palma de Mallorca**: Son Nebot, Escorca, 8 May 1950, F. Palau (MA 144131). **Salamanca**: Béjar, 24 Apr 1974, J. Aldasoro (MA 654066). **San Sebastián**: Bosque de Escoria, Herbarium Gredillae (MA 11222). **Santander**: Lomeña, 24 May 1984, Herrá & Moreno (MA 681498). **Segovia**: Aguilafuente, 1 May 1998, J.I. García Viñas & R. Alegría Delgado (MA 755044). **Sevilla**: Estepa, Pico Becerrero, 19 April 1968, S. Silvestre (SEV 106571). **Soria**: Santa Inés, 24 May 1958, A. Segura Zubizarreta (MA 355732). **Teruel**: Molino Nuevo de Arcos de las Salinas, 30 Apr 1988, G. Mateo (MA 440280). **Toledo**: between Hontanar and Cíjara, Estena River, 24 May 1968, E.F. Galiano et al. (SEV 106669). **Valencia**: Pico del Águila, Requena, May 1986, E. García (MA 382936). **Valladolid**: Tudela de Duero, Tovilla, 13 May 2006, L. Pascual (MA 758652). **Vitoria**: Vitoria, 1926, R. de Azua (MA 11223). **Zamora**: Cañizal, 10 May 1981, X. Giráldez (MA 311156). **Zaragoza**: Calatayud, 12 May 1906, C. Vicioso (MA 11201).

**16. *Poa ligulata*** Boiss.

**Total number of studied sheets: 295**. **SPAIN. Almería**: Bayárcal, Chullo, 5 May 1997, J. Lorite & R. Monterrubio (GDA 52369). **Burgos**: Humada, Peña Castro, 20 Jun 1987, M.L. Gil Zúñiga & J.A. Alejandre (MA 422994). **Cáceres**: suntrap of the Montfragüe castle, near Villareal de San Carlos, 20 May 1998, E. Fuertes (MA 796548). **Cádiz**: Grazalema, Sierra del Pinar, Puerto de las Palomas, 17 May 1991, V.E. Martín Osorio (MGC 33567). **Castellón**: to Sacañet, 15 Jun 1984, G. Mateo et al. (MA 465828). **Ciudad Real**: La Molata, 2 Jun 1934, J. González Albo (MA 11352). **Cuenca**: Ciudad Encantada, 13 May 1977, J. Fernández Díez et al. (SALA 11599). **Granada**: Montefrío, Íllora, Sierra de Parapanda, 18 May 2000, C. Morales et al. (GDA 42847). **Guadalajara**: N Guadalajara, 1 May 1965, B. Valdés (SEV 102927). **Jaén**: Cerro Cárceles, Sierra de Mágina, 18 May 2017, A. Ortega et al. (UNEX s.n.). **León**: Villarciño, 10 May 1984, C. Aedo (MA 619059). **Logroño**: Turruncún, Sierra de Préjano, Peña Isasa, 29 Jun 1988, F. Amich et al., Exsicata Selecta Florae Ibericae (SEV 234207). **Madrid**: Ontígola, 4 May 1974, S. Castroviejo (MA 644168). **Málaga**: Puerto del Oso, Ronda, 9 Jun 1988, A. Asensi et al. (MGC 24637). **Murcia**: Sierra de Villafuente, Fuente Cantalar, 17 May 2003, C. Aedo 9069 (MA 700826). **Navarra**: Sierra Urbasa, Monte Limitaciones, 15 Jun 1967, P. Montserrat 3345/67 (MA 216994). **Palencia**: Velilla del Río Carrión, 29 May 1987, M.E. García (MA 711393). **Santander**: Alto Muñata, Villosa de Ebro (Valdenedible), 25 Sep 1984, C. Aedo et al. (MA 681561). **Segovia**: Sepúlveda, 1 Jun 1986, X. Giraldez & T. Romero (SALA 41703). **Sevilla**: Algámitas, hill of Algámitas, 26 May 1972, B. Cabezudo & S. Silvestre (SEV 106694). **Soria**: Villabuena, 30 May 1970, A. Segura Zubizarreta (MA 355746). **Teruel**: Camarena de la Sierra, 10 Jun 1986, J.B. Peris & G. Stübing (MA 426115). **Toledo**: Noblejas, along the Tajo River, 18 Apr 2009, J. Calvo & S. Hantson JC3507 (MA 791041). **Valencia**: Apin jugum Moncabrer, Sierra Mariola, 11 Jun 1896, C. Pau (MA 11357). **Zaragoza**: Torralba de Ribota, 30 May 1977, S. Castroviejo et al. n. 715 SC (MA 247299).

**17. *Poa alpina*** L. subsp. ***alpina***

**Total number of studied sheets: 249 (**var. ***alpina*), 16 (**var. ***brevifolia*), 2 (**var. ***molinieri*) and 1 (**var. ***vivipara*)**.

var. ***alpina***. **ANDORRA.** Ascent from Grau-Roig to Stanis dels Pessons, 3 Jul 1992, C. Navarro CN-633 et al., Iter Andorrano-Aranense, VII-1992 (MA 525457).

**SPAIN. Barcelona**: Coll de Pal, 5 Jul 2003, S. Pyke et al. (BC 863721). **Bilbao**: high part of the Aldamín, 25 Jul 1948, E. Guinea (MA 835761). **Gerona**: Vall de Ribes, Clotada de la Fonseca, Queralbs, 21 Jul 1968, J. Vigo & A. Anglada (BC 601576). **Huesca**: Sierra de Oza, Valle de Guarrinza, 19 Jul 1980, J.A. Devesa et al. (SEV 99758). **León**: Puerto de las Señales, 29 Jun 1975, F. Llamas (SALA 93939). **Lérida**: Montarto, Naut Aran, 6 Aug 1984, A.M. Hernández (MA 541783). **Logroño**: Viniegra de Arriba, 11 Jun 1973, A. Segura Zubizarreta (MA 355705). **Navarra**: Valle del Roncal, 2 Aug 1987, M. Luceño et al. PV 2475 (MA 350575). **Oviedo**: Alto del Puerto de Somiedo, 30 Jun 1981, M.C. Fernández-Carvajal (MA 565233). **Palencia**: Agujas de Cardaño, Cardaño de Arriba, 11 Jul 1995, C. Aedo et al. CA 3618 (MA 560358). **Santander**: Peña Prieta, Vega de Liébana, 6 Aug 1985, C. Aedo (MA 619071). **Soria**: Santa Inés, Majadarrubia, 5 Aug 1975, A. Segura Zubizarreta (MA 355685). **Teruel**: Blancas, Mallo Leckerín suntrap, 20 Jul 1968, P. Montserrat 2970/68 (MA 211670). **Vitoria**: Lagrán, Monte Palomares, 25 Jul 1984, I.M. Icaya et al. (SEV 234199).

var. ***brevifolia*** (Gaudin) Godr. in Gren. & Godr. **ANDORRA.** Parish of La Massana, Alto de la Capa, 23 Jul 2005, C. Aedo et al. 12050 (MA 731555).

**SPAIN.** **Barcelona**: Coll de Pal, Tossa d'Alp, 5 Jul 2003, N. Ibáñez et al. (BC 863720). **Huesca**: Sallent de Gállego, Cdo. Musales and Respumoso, 9 Jul 1980, P. Montserrat & L. Villar (BC 950211). **Lérida**: Alta Ribagorça, Benasc, cabin of the Cabellud, 15 Aug 1975, J. Nuet 120J (BC 805953).

var. ***molinieri*** (Balb.) Endl. **ANDORRA.** Coll de Ondino, path of the Pic de Casamanya, 5 Jul 1992, G. Nieto Feliner 3252GN et al., Iter Andorrano-Aranense, VII-1992 (MA 514862).

**SPAIN.** **Lérida**: Clot del Munyidor, 25 Aug 2011, M. Guardiola & A. Petit (BC 877255).

**var. *vivipara*** L. **SPAIN. Navarra**: Valle del Roncal, Puerto de Arles, 27 Jul 1969, E.F. Galiano et al. (SEV 97164).
